# Preclinical targeting of liver fibrosis with a ^89^Zr-labeled Fibrobody® directed against platelet derived growth factor receptor-β

**DOI:** 10.1007/s00259-024-06785-9

**Published:** 2024-06-18

**Authors:** Joey A. Muns, Erik Schooten, Rychon D. J. van Dasselaar, Yvet E. Noordman, Kevin Adamzek, Arthur C. Eibergen, Sebas D. Pronk, Sagel Cali, Niels J. Sijbrandi, Eugen Merkul, Sabrina Oliveira, Paul M.P. van Bergen en Henegouwen, R. Bart Takkenberg, Joanne Verheij, Stan F.J. van de Graaf, Bart A. Nijmeijer, Guus A.M.S. van Dongen

**Affiliations:** 1 LinXis Biopharmaceuticals, Amsterdam, the Netherlands; 2 Department of Biology, Division of Cell Biology, Neurology and Biophysics, Science Faculty, Utrecht University, Utrecht, the Netherlands; 3 Department of Pharmaceutical Sciences, Pharmaceutics Devision, Faculty of Science, Utrecht University, Utrecht, the Netherlands; 4 Department of Gastroenterology and Hepatology, Amsterdam UMC, Amsterdam, the Netherlands; 5 Department of Pathology, Amsterdam UMC, Amsterdam, the Netherlands; 6 Tytgat Institute for Liver and Intestinal Research, Amsterdam University Medical Centers, University of Amsterdam, Amsterdam, The Netherlands; 7 Amsterdam Gastroenterology, Endocrinology and Metabolism (AGEM), Amsterdam University Medical Centers, Amsterdam, The Netherlands; 8 Department of Radiology and Nuclear Medicine, Amsterdam UMC location Vrije Universiteit Amsterdam, Amsterdam, the Netherlands

**Keywords:** Liver fibrosis, PDGFRβ, Activated myofibroblasts, ^89^Zr-immuno-PET, Fibrobody®, Tracer and drug conjugates

## Abstract

**Purpose:**

Hepatic fibrosis develops as a response to chronic liver injury, resulting in the formation of fibrous scars. This process is initiated and driven by collagen-producing activated myofibroblasts which reportedly express high levels of platelet derived growth factor receptor-β (PDGFRβ). We therefore regard PDGFRβ as an anchor for diagnosis and therapy. The Fibrobody® SP02SP26-ABD is a biparatopic VHH-construct targeting PDGFRβ. Here, we explore its potential as a theranostic vector for liver fibrosis.

**Methods:**

Specificity, cross-species binding, and cellular uptake of SP02SP26-ABD was assessed using human, mouse and rat PDGFRβ ectodomains and PDGFRβ-expressing cells. Cellular uptake by PDGFRβ-expressing cells was also evaluated by equipping the Fibrobody® with auristatinF and reading out in vitro cytotoxicity. The validity of PDGFRβ as a marker for active fibrosis was confirmed in human liver samples and 3 mouse models of liver fibrosis (DDC, CCl_4_, CDA-HFD) through immunohistochemistry and RT-PCR. After radiolabeling of DFO*-SP02SP26-ABD with ^89^Zr, its in vivo targeting ability was assessed in healthy mice and mice with liver fibrosis by PET-CT imaging, ex vivo biodistribution and autoradiography.

**Results:**

SP02SP26-ABD shows similar nanomolar affinity for human, mouse and rat PDGFRβ. Cellular uptake and hence subnanomolar cytotoxic potency of auristatinF-conjugated SP02SP26-ABD was observed in PDGFRβ-expressing cell lines. Immunohistochemistry of mouse and human fibrotic livers confirmed co-localization of PDGFRβ with markers of active fibrosis. In all three liver fibrosis models, PET-CT imaging and biodistribution analysis of [^89^Zr]Zr-SP02SP26-ABD revealed increased PDGFRβ-specific uptake in fibrotic livers. In the DDC model, liver uptake was 12.15 ± 0.45, 15.07 ± 0.90, 20.23 ± 1.34, and 20.93 ± 4.35%ID/g after 1,2,3 and 4 weeks of fibrogenesis, respectively, compared to 7.56 ± 0.85%ID/g in healthy mice. Autoradiography revealed preferential uptake in the fibrotic (PDGFRβ-expressing) periportal areas.

**Conclusion:**

The anti-PDGFRβ Fibrobody® SP02SP26-ABD shows selective and high-degree targeting of activated myofibroblasts in liver fibrosis, and qualifies as a vector for diagnostic and therapeutic purposes.

**Supplementary Information:**

The online version contains supplementary material available at 10.1007/s00259-024-06785-9.

## Introduction

Hepatic fibrosis is a dynamic process characterized by the excessive accumulation of extracellular matrix proteins as a result of chronic liver injury caused by a diversity of etiologies, including viral infections, alcoholic liver disease, and nonalcoholic fatty liver disease (NAFLD). In 2020 the term metabolic dysfunction-associated steatotic liver disease (MASLD) was introduced as it better reflects NAFLD related to the metabolic syndrome, which is characterized by obesity, type 2 diabetes mellitus, and dyslipidemia [1]. The more advanced stage of NAFLD/MASLD, coined non-alcoholic steatohepatitis (NASH), respectively metabolic dysfunction-associated steatohepatitis (MASH), can lead to fibrosis, cirrhosis and liver failure. In addition, these conditions increase the risk of developing hepatocellular carcinoma. As the prevalence of obesity is rapidly rising, so is the prevalence of MASH [2, 3]. The stage of fibrosis has consistently been shown to be the best predictor of survival and quality of life in these patients [4, 5]. At tissue level, hepatic fibrosis develops as a response to chronic hepatotoxicity or cholestatic injury, resulting in inflammatory responses, differentiation of, amongst others, quiescent hepatic stellate cells (HSCs) into activated, collagen-producing myofibroblasts, accumulation of extracellular matrix proteins, and formation of fibrous scars [6].

Today, liver fibrosis is mostly diagnosed at an advanced stage, when clinical signs first become apparent. The gold standard for assessing fibrosis is liver biopsy, which has intrinsic limitations related to invasiveness and patient safety, and nonrepresentative sampling. Therefore, efforts have been made to develop non-invasive tests, such as radiological tests like transient elastography for assessing liver stiffness (e.g. Fibroscan), serum biomarker assays (e.g. FibroTest), or multifactorial scoring systems [7, 8]. Unfortunately, these methods are still limited by their variable sensitivity and specificity, and especially by their poor ability to discriminate active from dormant disease. Lack of reliable diagnostic tests also hampers the development of new therapeutic drugs as far as patient selection and response monitoring in clinical therapy trials concerns.

Prevention and treatment of MASLD includes weight loss through dietary and exercise programs, while most recently new antidiabetic drugs that induce weight loss are attracting attention [9, 10]. However, weight loss may be difficult to achieve and maintain, and effect on resolution of advanced stage MASH has yet to be demonstrated. Therefore, as an alternative, several drugs acting on cellular and molecular mechanisms of fibrosis have been tested for treatment of NASH/MASH [11]. Unfortunately, no drugs have been approved yet by the Food and Drug Administration (FDA), which considers MASH with fibrosis or cirrhosis an area of unmet clinical need [8].

While fibrotic diseases can arise in many organs in response to (chronic) organ injury and stress, and collectively form a major global healthcare burden with a contribution to 45% of all-cause mortality world-wide, drug development in this field thus far has been of limited success [12]. The only antifibrotics approved so far are nintedanib and pirfenidone, both for patients with interstitial pulmonary fibrosis. The majority of treatment strategies under development are limited to individual fibrotic diseases or organs associated with high morbidity or mortality, focusing on initial steps leading to fibrogenesis such as organ cell death, inflammation, oxidative stress and imbalanced metabolism. Nevertheless, many of these treatment strategies are hampered by patient group heterogeneity and the rare incidence of many fibrotic disorders.

A potential alternative strategy to overcome aforementioned challenges is to follow a pan-fibrosis approach by targeting common driver cells and molecular pathways which are shared during progression across different fibrotic diseases in different organs [13–15]. One such common feature is the switch of resident fibroblasts leading to activated myofibroblasts [6, 16–19]. These activated myofibroblasts are the central drivers of fibrogenesis and responsible for ongoing accumulation of extracellular matrix proteins, matrix remodeling and production of proinflammatory and chemoattractant mediators. In addition, myofibroblasts express high levels of alpha smooth muscle actin (α-SMA) and have proliferative, motile, and contractile properties. Their constriction can affect physiological tissue architecture, impairing blood flow, nutrient exchange and organ function especially in advanced stage fibrosis. Therefore, activated myofibroblasts might be attractive cells for high-precision therapeutic interventions across different fibrotic diseases [14, 20–25].

As a theranostic pan-fibrosis approach we aim for selective targeting, detection and silencing of myofibroblasts via cell-selective anchors. For this purpose, we selected platelet derived growth factor receptor-β (PDGFRβ) as a lead candidate anchor, as this surface receptor is reportedly over-expressed on activated myofibroblasts, with limited expression on healthy tissues [26–30]. We employ ^89^Zr-immuno-PET as introduced by our group, for the navigation and de-risking of the Fibrobody® platform during preclinical and clinical development [31].

We generated a Fibrobody® construct, designated SP02SP26-ABD, which is based on variable fragments of heavy chain-only antibodies (VHH) designed for selective targeting of PDGFRβ. In the choice for and the design of the Fibrobody® the next considerations were followed: (1) a biparatopic design to optimize internalization and to allow species cross-reactivity (human, mouse, rat) at similar affinity to facilitate clinical translation, (2) presence of an albumin binding domain (ABD) to avoid rapid renal clearance and excessive kidney accumulation [32], (3) presence of a near C-terminal cysteine group to allow site-specific conjugation of diagnostic or therapeutic payloads, (4) absence of agonistic activity, (5) small size to facilitate tissue penetration and (6) absence of an Fc region to exclude interaction with the immune system.

In the present study, we have evaluated the PDGFRβ-binding Fibrobody® SP02SP26-ABD, against the non-binding control construct R2R2-ABD, for its potential of selective targeting of liver fibrosis. The specificity, cross-species binding characteristics and internalizing ability of SP02SP26-ABD were investigated. After radiolabeling with ^89^Zr, the biodistribution of [^89^Zr]Zr-SP02SP26-ABD was evaluated in three different mouse models of liver fibrosis.

## Methods and materials

### Cell lines

The mouse squamous cell carcinoma VII (SCC) cell line was kindly provided by Prof. dr. Maria C. Pedroso de Lima, University of Coimbra, Coimbra, Portugal. SCC cells were stably transfected with in-house generated expression vectors encoding for human, mouse or rat PDGFRβ fused to GFP at the c-terminal end (SCC-hPDGFRβ, SCC-mPDGFRβ and SCC-rPDGFRβ, respectively), and clones were sorted for high GFP signal by FACS. The cell lines were cultured in Dulbecco’s Modified Eagle’s Medium (DMEM) (Lonza, Basel, Switzerland) with 100 units/mL streptomycin, 0.1 mg/mL penicillin, and 10% fetal bovine serum (FBS). To maintain transgene expression, the cells were kept under selection pressure with 400 µg/mL G418 Sulfate (Thermo Fisher Scientific, Bleiswijk, the Netherlands). Cells were kept at 37 °C in a humidified atmosphere containing 5% CO_2_ and were repeatedly tested negative for mycoplasm.

### Fibrobody® SP02SP26-ABD and control construct R2R2-ABD

Information on the generation and production of SP02SP26-ABD and R2R2-ABD can be found in the supplementary information.

### Fibrobody® conjugates

The near C-terminal cysteine in the SP02SP26-ABD and the R2R2-ABD constructs was used for site-specific maleimide-IRDye800CW, maleimide-auristatinF, maleimide-DFO* and maleimide-ethyl conjugation. The chelator DFO* was conjugated to allow radiolabeling with ^89^Zr. For some assays, an ethyl group was conjugated to avoid dimerization due to the reduced cysteine. Details on conjugation conditions, purification of the conjugates, radiolabeling and the quality testing of the conjugates can be found in the supplementary information.

### Binding to PDGFRβ ectodomain and SCC-PDGFRβ cells

Procedures to assess binding of SP02SP26-ABD and R2R2-ABD to human, mouse or rat PDGFRβ ectodomain and transfected SCC cells are described in the supplementary information.

### Cellular uptake assay

SCC-hPDGFRβ, SCC-mPDGFRβ or SCC-rPDGFRβ cells (approximately 5.000 cells per well) were seeded in a 96-well tissue culture plate (Nunclon® Delta Surface, Thermo Fisher Scientific, Bleiswijk, The Netherlands), 48 h prior to the assay. Uptake of SP02SP26-ABD-IRDye800CW and R2R2-ABD-IRDye800CW (20 nM) by cells was measured during incubation at 37 °C for a period of 60 min. Internalization was stopped by placing cells onto ice. After two sequential washes with PBS, the surface fraction was removed from the cells by two sequential acid washes (0.2 M Glycine-HCl, 150 mM NaCl, pH 2.3), and fluorescence was measured (cellular uptake).

### Cell viability assays

As starting point, 500 SCC, SCC-hPDGFRβ, SCC-mPDGFRβ or SCC-rPDGFRβ cells were seeded in flat bottom 96-well tissue culture treated plates (Nunclon® Delta Surface, Thermo Fisher Scientific, Bleiswijk, The Netherlands) one day prior to the assay. Next day, four-fold serial dilutions from auristatinF-conjugated VHH constructs, starting from 40 nM, were added onto the target cells and incubated for 5 days at 37 °C. After 5 days, Cell-Titer-Blue® (CTB) reagent (Promega, Leiden, The Netherlands) was added and incubated for 4 h at 37 °C. Then, the reaction was stopped upon addition of 3% SDS and fluorescence at 590 nm was measured on a FLUOstar OPTIMA FL microplate reader (BMG LABTECH Ortenberg, Germany). Results were analyzed using GraphPad Prism software.

### Animal studies and fibrotic liver models

Animal experiments were performed according to the NIH Principles of Laboratory Animal Care, the European Community Council Directive (2010/63/EU) for laboratory animal care and the Dutch Law on animal experimentation (“Wet op de dierproeven”, Stb 1985, 336). The experimental protocol was validated and approved by the central Dutch national committee for animal experimentation (CCD) and the local committee on animal experimentation of the Amsterdam UMC, Vrije Universiteit Amsterdam. Studies were conducted in C57Bl/6 N mice. Animals were obtained from Charles River (Sulzfeld, Germany), aged 8–10 weeks, left for at least two weeks to acclimatize, after which they were allocated to the experimental groups.

DDC model [33]: The chemical substance 3,5-diethoxycarbonyl-1,4-dihydrocollidine (DDC, Sigma, Houten, The Netherlands) was admixed to the food. Ingestion of DDC results in cholestasis, which in turn causes (cholestatic) liver fibrosis. To this end, female control mice received C1000 rodent diet (Altromin, Lage, Germany) as standard chow, while female DDC mice received C1000 enriched with 0.05% DDC (Triple A Trading, Tiel, The Netherlands) for a period up to 4 weeks. Both diets contain 80 g/kg maltodetrin and 110 g/kg cream to increase palatability.

CCl_4_ model [34]: The hepatoxic chemical carbon tetrachloride (CCl_4_, Sigma, St. Louis, MO, USA) was repeatedly injected intraperitoneal (i.p.), resulting in chemical-induced liver fibrosis. To this end, female mice received injections of CCl_4_ in corn oil (Sigma, St. Louis, MO, USA) (1:3, v: v) twice weekly for a period of 4 weeks. The volume of each injection was 50 µL per 25 gram body weight, resulting in 0.5 uL CCl_4_ per gram body weight.

CDA-HFD model [35]: In the choline-deficient L-amino acid-defined high-fat diet (CDA-HFD) MASLD model, an L-amino acid diet with 60 kcal% fat with 0.1% methionine and no choline (Research Diets Inc, New Brunswick, NJ, USA) was used for a period of 6 weeks to induce liver fibrosis.

### PET-CT imaging, ex vivo biodistribution and autoradiography

Mice were intravenously (i.v.) injected via the retro-orbital plexus with 1 MBq of [^89^Zr]Zr-SP02SP26-ABD or [^89^Zr]Zr-R2R2-ABD and scanned 24 h post-injection using a dedicated small animal NanoPET/CT (Mediso Ltd., Hungary). Each animal received mass doses based on its individual weight. Mice were anaesthetized during the whole scanning period (1 h duration) by inhaling 2% isoflurane/O_2_. A 5 min CT scan was acquired for attenuation and scatter correction purposes. Reconstruction was performed using a 3-dimensional reconstruction algorithm (Tera-Tomo; Mediso Ltd.) with four iterations and six subsets, resulting in an isotropic 0.4 mm voxel dimension.

Immediately after the PET-CT scanning procedure, all mice (3–4 mice per group) were anaesthetized, bled, euthanized and tissues harvested for assessment of the ex vivo biodistribution, autoradiography, (immuno)histochemistry and RT-PCR. For all mice, blood and organs of interest were collected, weighed and the amount of radioactivity in each sample was measured in an Automatic Gamma Counter (HIDEX, Turku, Finland). Radioactive uptake was calculated as percentage of injected dose per gram of tissue (%ID/g).

For autoradiography, livers were flash-frozen in liquid nitrogen cooled isopentane. A cryostat-microtome was used to cut the frozen livers in 8 μm sections, which were mounted on adhesive glass slides. Sections were exposed for 1–2 weeks to a phosphor screen BAS-IP SR 2040 E (General Electric, Eindhoven, The Netherlands). After exposure, plates were scanned using a Typhoon FLA 7000 imager (General Electric, Eindhoven, The Netherlands).

### (Immuno)histochemistry and RT-PCR

Procedures for (immuno)histochemistry (IHC) and RT-PCR have been described in the supplementary information.

### Serum markers for liver damage

See supplementary information.

### Statistics

The Grubbs outlier test was used to check and remove outliers and statistical analysis was performed on the tissue uptake values of the different groups of mice with the Welch’s T-test for paired data. Two-sided significance levels were calculated and *p* < 0.05 was considered to be statistically significant. All graphs were generated using GraphPad Prism 9 software.

## Results

### PDGFRβ binding and cellular uptake of SP02SP26-ABD

Generation of the biparatopic SP02SP26-ABD construct will be described elsewhere, and herein the most important binding and internalization characteristics will be described.

In SP02SP26-ABD and the non-binding control construct R2R2-ABD, the VHH units are linked with a short glycine-serine rich linker (G_4_S)_3_, while the constructs contain an albumin binding domain (ABD) as a half-life extension unit for in vivo applications. For site-specific conjugation of fluorophore, chelator or drug, a cysteine was engineered at the C-terminal region together with an EPEA tag to facilitate purification. The molecular weight of the total constructs is ∼ 36 kDa.

**Fig. 1:**
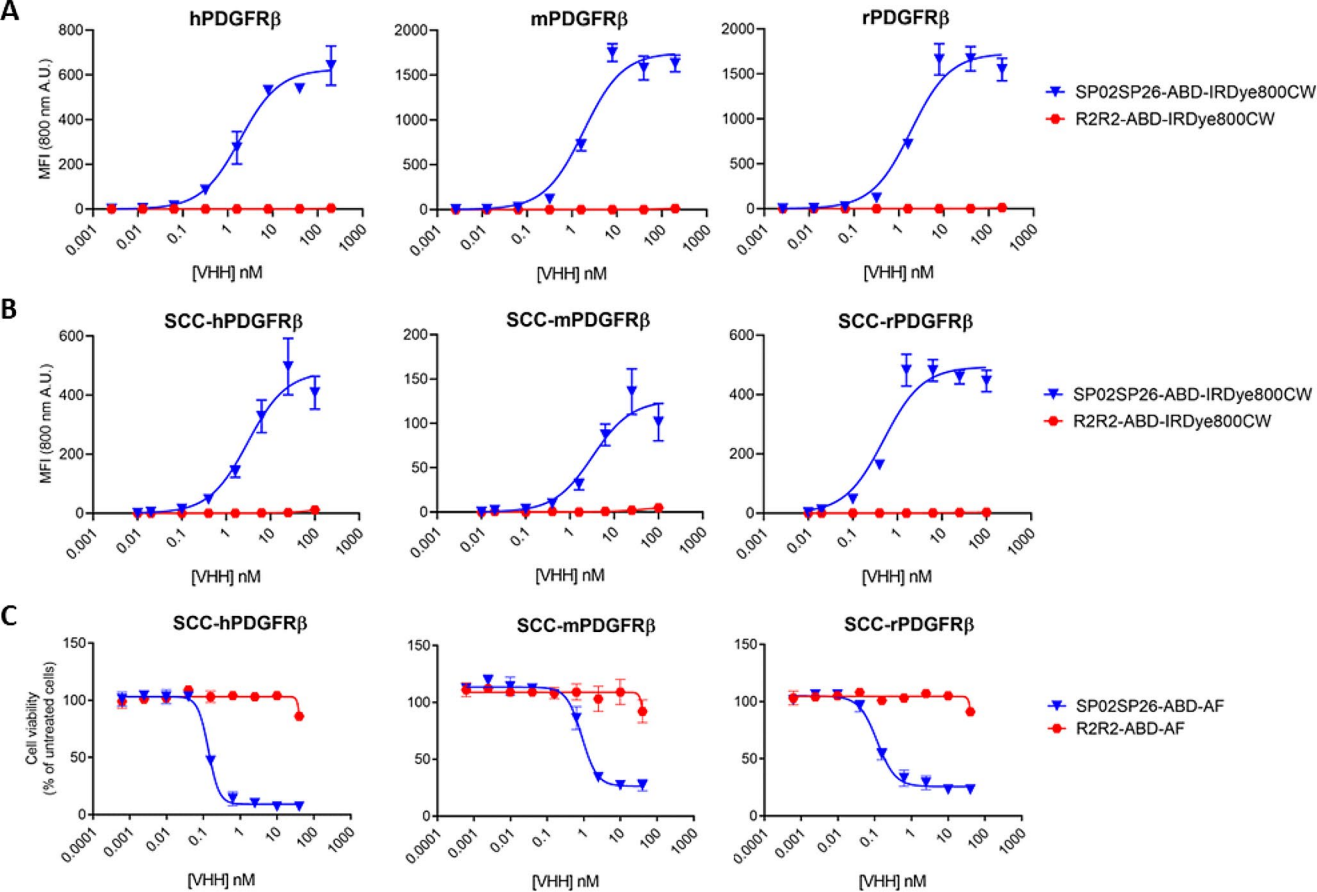
PDGFRβ binding and drug delivery characteristics of SP02SP26-ABD. (**A**) Binding of SP02SP26-ABD and non-binding control R2R2-ABD, both conjugated to IRDye800CW, to human, mouse, and rat PDGFRβ ectodomain, and (**B**) to SCC-hPDGFRβ, SCC-mPDGFRβ and SCC-rPDGFRβ cells. (**C**) Cell viability of SCC-hPDGFRβ, SCC-mPDGFRβ and SCC-rPDGFRβ cells upon treatment with SP02SP26-ABD-AF and R2R2-ABD-AF. Non-transfected SCC cells also have been tested in B and C, and neither showed binding nor decreased viability (data not shown). MFI = Median Fluorescence Intensity

To illustrate the species cross-reactivity of SP02SP26-ABD, binding studies were performed with IRDye800CW-conjugated SP02SP26-ABD and R2R2-ABD on recombinant ectodomains of human, mouse and rat PDGFRβ (h-, m- and rPDGFRβ, respectively) (Fig. 1A). Binding of SP02SP26-ABD-IRDye800CW occurred at similar affinity, with K_D_ values being 1.9 ± 0.3, 1.8 ± 0.4 and 1.9 ± 0.4 nM, respectively, while no binding was observed with R2R2-ABD-IRDye800CW. Similar affinities were observed when PDGFRβ-transfected SCC cells were used for binding: K_D_ values were 3.1 ± 0.7, 3.3 ± 1.0 and 0.5 ± 0.1 nM for the cell lines SCC-hPDGFRβ, SCC-mPDGFRβ and SCC-rPDGFRβ (Fig. 1B), while no binding with the parental SCC cell line was observed (data not shown). In addition, lack of cross-reactivity of SP02SP26-ABD with human and mouse PDGFRα was demonstrated (Supplementary Fig. 1). The affinity of SP02SP26-ABD-IRDye800CW for binding to human, mouse and rat serum albumin appeared to be 5.2, 10.0 and 1.7 nM, respectively (Supplementary Fig. 2). SP02SP26-ABD-IRDye800CW showed uptake in all 3 PDGFRβ-transfected cell lines after a 1 h incubation period, while this was not the case for the control construct R2R2-ABD-IRDye800CW (Supplementary Fig. 3). To confirm these results, and to obtain in vitro proof of concept for intracellular drug delivery by the Fibrobody®, SP02SP26-ABD and R2R2-ABD were conjugated to the very potent cytotoxic compound auristatinF (AF) and the conjugates were evaluated in cell viability assays (Fig. 1C). SP02SP26-ABD-AF showed effective killing of all three PDGFRβ-transfected cell lines, with comparable IC_50_ values for SCC-hPDGFRβ, SCC-mPDGFRβ and SCC-rPDGFRβ of 0.10 ± 0.05, 0.90 ± 0.04 and 0.10 ± 0.05 nM, respectively, while no effective killing of the non-transfected SCC cell line (data not shown) or with R2R2-ABD-AF was observed.

Finally, to investigate whether SP02SP26-ABD possesses intrinsic agonistic activity, SCC-hPDGFRβ cells were incubated with SP02SP26-ABD and lysed to evaluate phosphorylation of PDGFRβ. As a positive control for PDGFRβ activation, its natural ligand PDGF-BB was used. Supplementary Fig. 4 shows that SP02SP26-ABD did not activate the PDGFRβ receptor, while PDGF-BB did.

### Binding of SP02SP26-ABD to human fibrotic liver sections

**Fig. 2:**
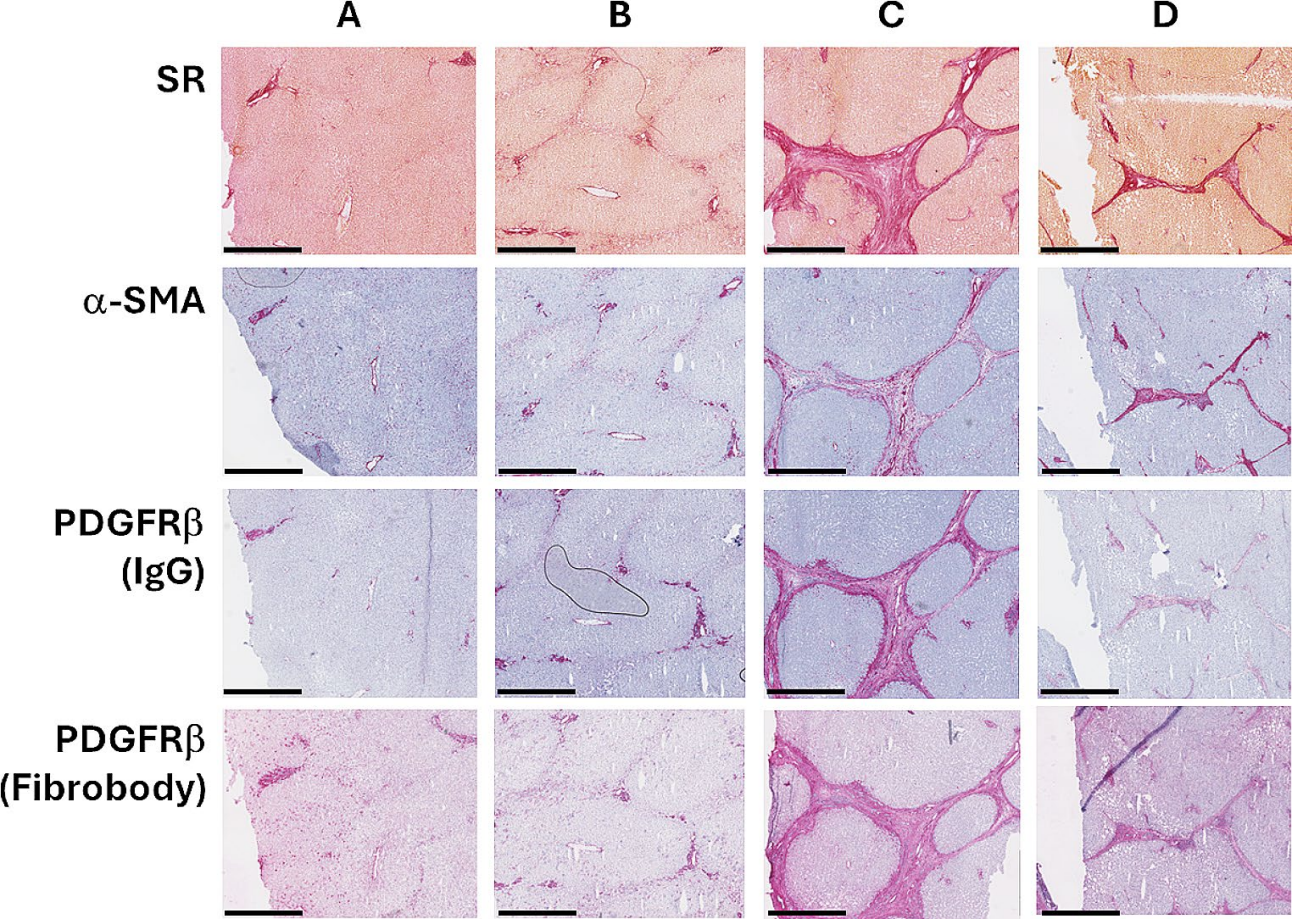
(Immuno)histochemical analysis of human liver tissue at various fibrotic stages. Top row: (Picro)Sirius Red (SR). Second row: α-SMA. Third row: PDGFRβ by commercial IgG and fourth row: PDGFRβ by Fibrobody®. Left to right: (**A**) Normal liver. (**B**) Early periportal fibrosis secondary to biliary compression due to adjacent adenoma. (**C**) Active cirrhosis secondary to autoimmune hepatitis. (**D**) Extinguished cirrhosis secondary to HCV infection, 10 years after start of antiviral pharmacotherapy. 3.5-fold digital zoom. Black bar in the bottom signifies 1 mm. Data acquired via screenshots of digital scans, using NDP.view2 software (Hamamatsu Photonics, Hamamatsu, Japan)

Cryosections of human fibrotic liver were (immuno)histochemically stained (1) to show staining of hPDGFRβ with SP02SP26-ethyl next to staining with a commercially available anti-PDGFRβ IgG antibody and of other fibrosis markers, and (2) to show staining of hPDGFRβ at different stages of liver fibrosis. A similar staining pattern was observed for both PDGFRβ binding ligands (Fig. 2). Moreover, within this small series, the extent of staining appeared to be related to fibrosis activity.

### PDGFRβ targeting with [^89^Zr]Zr-SP02SP26-ABD in the DDC liver fibrosis model

As a first step in the in vivo evaluation of SP02SP26-ABD, the 3,5-diethoxycarbonyl-1,4-dihydrocollidine (DDC) cholestatic model of liver fibrosis was used. The DDC diet resulted in a slight weight loss (∼ 10%) after the start of the diet, and increased concentrations of alkaline phosphatase (AP), alanine aminotransferase (ALT) and aspartate aminotransferase (AST) were observed in plasma (Supplementary Fig. 5). The model was characterized for the expression of fibrosis markers, PDGFRβ included, after feeding mice a 0.05% DDC enriched chow (DDC diet) for different time intervals. The targeting characteristics of 15 nmol/kg [^89^Zr]Zr-SP02SP26-ABD were assessed at the end of these intervals.

**Fig. 3:**
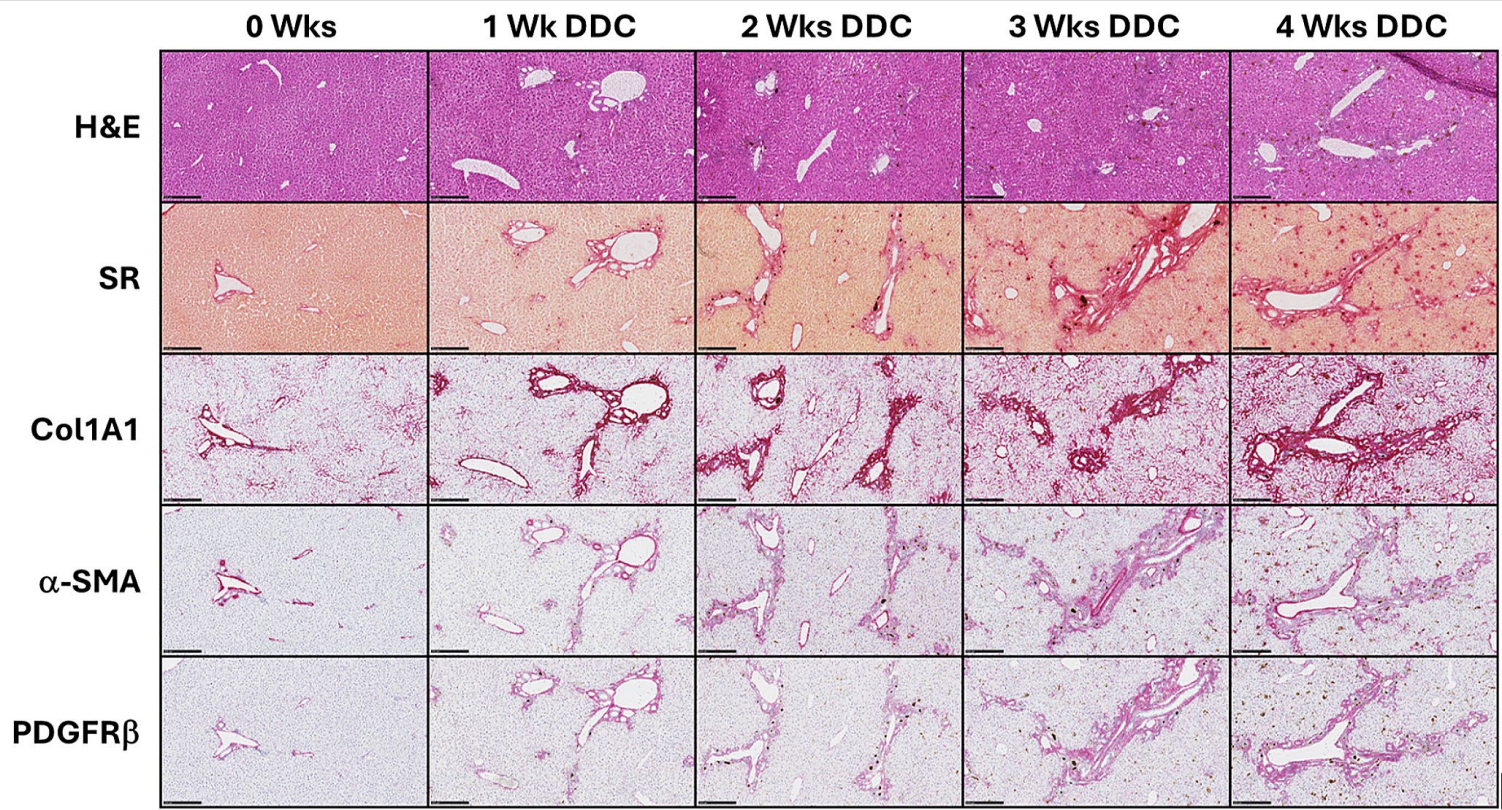
(Immuno)histochemistry of liver tissues of mice on control diet (0 group), or 1, 2, 3 or 4 weeks on DDC diet. Results of representative animals (10x digital overview, same animal top to bottom). H&E: Haematoxylin-Eosin. SR: (Picro)Sirius Red. Black bar in the bottom signifies 250 μm. Data acquired via screenshots of digital scans, using NDP.view2 software (Hamamatsu Photonics, Hamamatsu, Japan)

Increased expression of a panel of fibrosis related genes was confirmed by RT-PCR (Table 1). In addition, IHC of livers revealed increased expression of PDGFRβ after mice had been fed DDC diet for 1 week, while expression further increased after 2, 3 and 4 weeks of DDC diet (Fig. 3). Increased expression of PDGFRβ was spatially accompanied by increased α-SMA expression and collagen deposition, the latter being visualized by (picro)sirius red and Col1A1 staining.

**Table 1:** Expression of fibrosis related genes as assessed by RT-PCR in liver tissues of mice on control diet (healthy controls), or 1, 2, 3 or 4 weeks on DDC diet

Transcript	Week 1 DDC	Week 2 DDC	Week 3 DDC	Week 4 DDC
Pdgfrβ	+	+	+	+
Col1a1	+++	+++	+++	+++
Col1a2	++	++	++	++
Col3a1	++	++	++	++
a-Sma	++	+	++	++
Pdgfrα	+	+	+	+
Timp1	+++	+++	+++	+++
Lox	+++	+++	+++	++
Tgfβ1	+	+	+	+

-: not upregulated, +: 1–5 fold upregulated, ++: 5–20 fold upregulated, +++>20 fold upregulated in mRNA isolated from liver homogenates, as compared to healthy controls

**Fig. 4:**
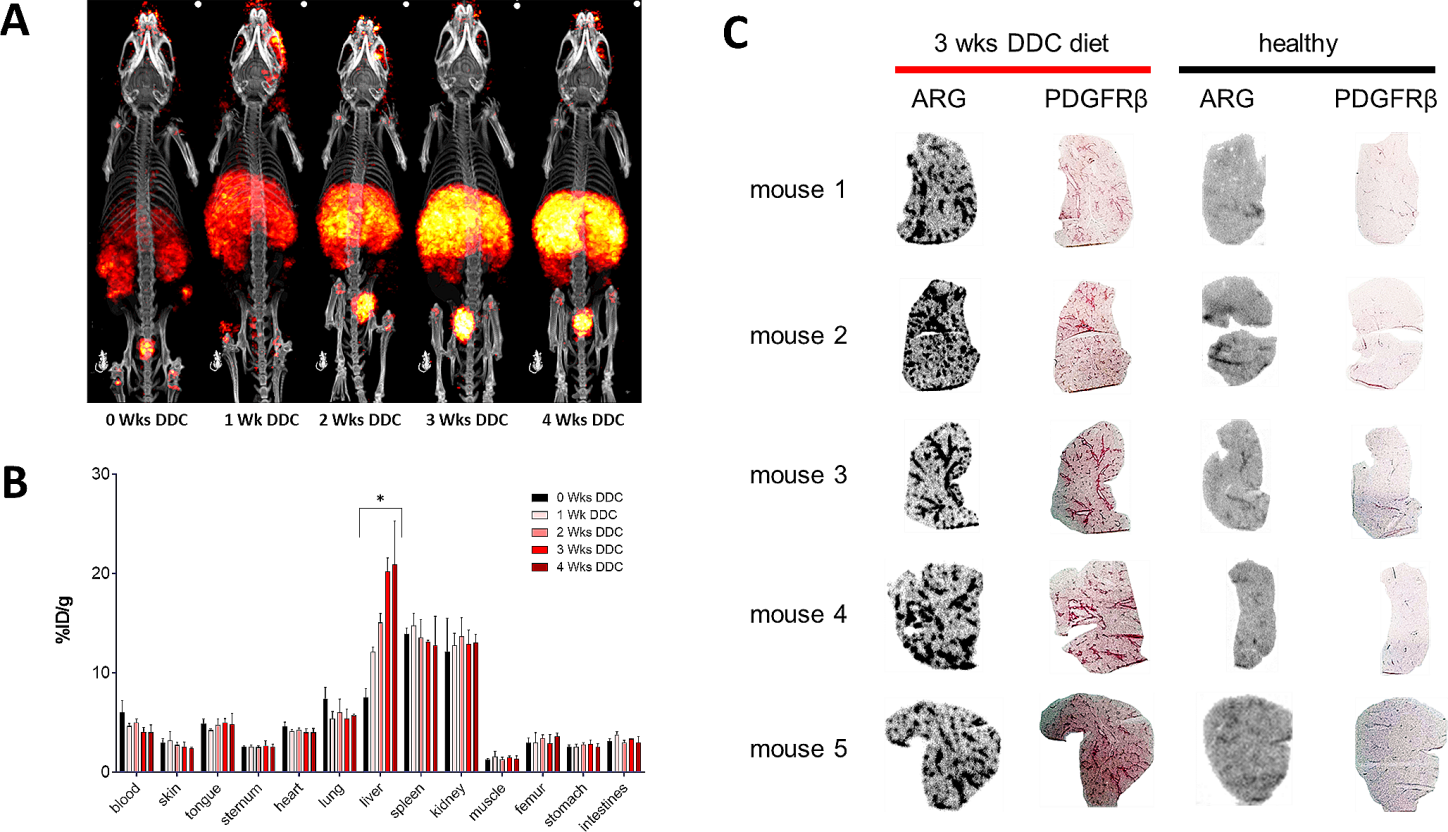
PET-CT scans, ex vivo biodistribution and ex vivo autoradiography (ARG) of [^89^Zr]Zr-SP02SP26-ABD in mice with liver fibrosis. (**A**) Representative scans of control mice (0 weeks group, healthy mice) and of mice with liver fibrosis after 1, 2, 3, or 4 weeks on DDC diet, at the end of DDC intake, 24 h after injection of 15 nmol/kg [^89^Zr]Zr-SP02SP26-ABD. All scans were scaled to reflect the same injected ^89^Zr dose. (**B**) Ex vivo biodistribution. Mean ± SD, *n* = 3 per group. Welsh t-test resulted in following *p*-values in liver; 0 wks DDC vs. 1 wk DDC *p* = 0.0036; 0 wks vs. 2 wks, *p* = 0.00046; 0 wks vs. 3 wks, *p* = 0.00082; 0 wks vs. 4 wks, *p* = 0.035; 1 wk vs. 2 wks, *p* = 0.015; 1 wk vs. 3 wks, *p* = 0.010; 1 wk vs. 4 wks, *p* = 0.074; 2 wks vs. 3 wks, *p* = 0.012; 2 wks vs. 4 wks, *p* = 0.15; 3 wks vs. 4 wks, *p* = 0.81. *p*-value < 0.05 was considered to be significant. (**C**) ARG results are of liver sections of 5 individual mice of the 3 weeks DDC diet group and the control group, next to immunohistochemical staining for PDGFRβ on sequential sections

PET-CT imaging studies at the end of the DDC diet period showed a gradual increase in liver uptake of [^89^Zr]Zr-SP02SP26-ABD upon longer DDC diet time, reflecting the increased expression of PDGFRβ (Fig. 4A). Ex vivo biodistribution analysis confirmed an increased liver uptake (Fig. 4B). While uptake of [^89^Zr]Zr-SP02SP26-ABD in healthy livers of control mice was 7.56 ± 0.85%ID/g, this was 12.15 ± 0.45, 15.07 ± 0.90, 20.23 ± 1.34, and 20.93 ± 4.35%ID/g for fibrotic livers of mice on DDC diet for 1, 2, 3 and 4 weeks, respectively. Among healthy organs, relatively high uptake was also observed in spleen and kidney, but in these organs uptake of [^89^Zr]Zr-SP02SP26-ABD remained very similar during the period of DDC intake.

To evaluate intra-liver distribution of [^89^Zr]Zr-SP02SP26-ABD, livers of control mice and mice that received 3 weeks of DDC diet were cryo-sectioned and processed for autoradiography, while sequential sections were stained for PDGFRβ expression. Uptake of [^89^Zr]Zr-SP02SP26-ABD in fibrotic livers is particularly high in the periportal areas with PDGFRβ-positive cells (Fig. 4C).

**Fig. 5:**
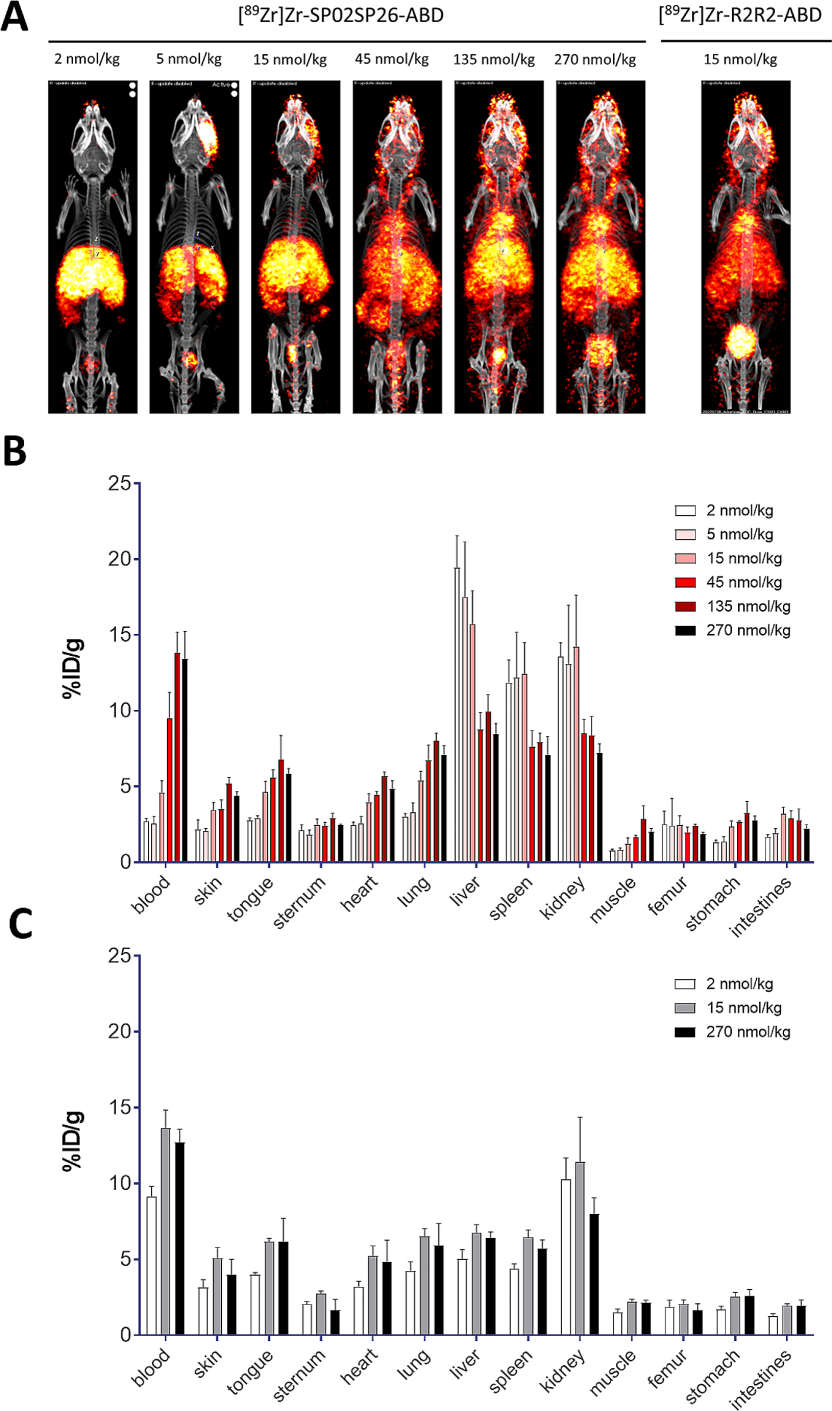
PET-CT scans and ex vivo biodistribution of [^89^Zr]Zr-SP02SP26-ABD and control construct [^89^Zr]Zr-R2R2-ABD in mice with liver fibrosis after 3 weeks on DDC diet. (**A**) Representative scans at the end of DDC intake, 24 h after injection of 2, 5, 15, 45, 135 and 270 nmol/kg [^89^Zr]Zr-SP02SP26-ABD (left panel), or 15 nmol/kg [^89^Zr]Zr-R2R2-ABD (right panel). All scans were scaled to reflect the same injected ^89^Zr dose (**B**) Ex vivo biodistribution of [^89^Zr]Zr-SP02SP26-ABD. (**C**) Ex vivo biodistribution of [^89^Zr]Zr-R2R2-ABD. Mean ± SD, *n* = 3 per group

To assess the Fibrobody® dose dependency of fibrosis targeting, the biodistribution of 2, 5, 15, 45, 135 and 270 nmol/kg [^89^Zr]Zr-SP02SP26-ABD was evaluated in mice with overt liver fibrosis (3 weeks DDC diet), 24 h after i.v. injection of the tracer (Fig. 5A). For comparison, the control construct [^89^Zr]Zr-R2R2-ABD was evaluated at 2, 15 and 270 nmol/kg. PET-CT imaging revealed high and selective uptake of [^89^Zr]Zr-SP02SP26-ABD in fibrotic livers, this in contrast to [^89^Zr]Zr-R2R2-ABD. At concentrations > 45 nmol/kg, next to uptake in the fibrotic liver region, also presence in the heart region and the carotids became apparent, indicative for circulating radioimmunoconjugate. Ex vivo biodistribution analysis revealed the highest uptake in the fibrotic liver of 19.44 ± 2.12%ID/g at a 2 nmol/kg dose, which gradually decreased and plateaued at higher doses: liver uptake at 5, 15, 45, 135 and 270 nmol/kg was 17.51 ± 3.64, 15.79 ± 1.85, 8.75 ± 1.13, 9.97 ± 1.10 and 8.49 ± 0.67%ID/g, respectively (Fig. 5B). In the same Fibrobody® dose range, decreased liver uptake was accompanied by higher blood levels, indicating PDGFRβ target saturation in the fibrotic liver. As expected, uptake of [^89^Zr]Zr-R2R2-ABD in the fibrotic liver was at a similar low level for the 2, 15 and 270 mmol/kg groups (5.02 ± 0.63, 6.83 ± 0.34, 6.41 ± 0.40%ID/g, respectively).

### Kinetics of [^89^Zr]Zr-SP02SP26-ABD in DDC liver fibrosis model

**Fig. 6:**
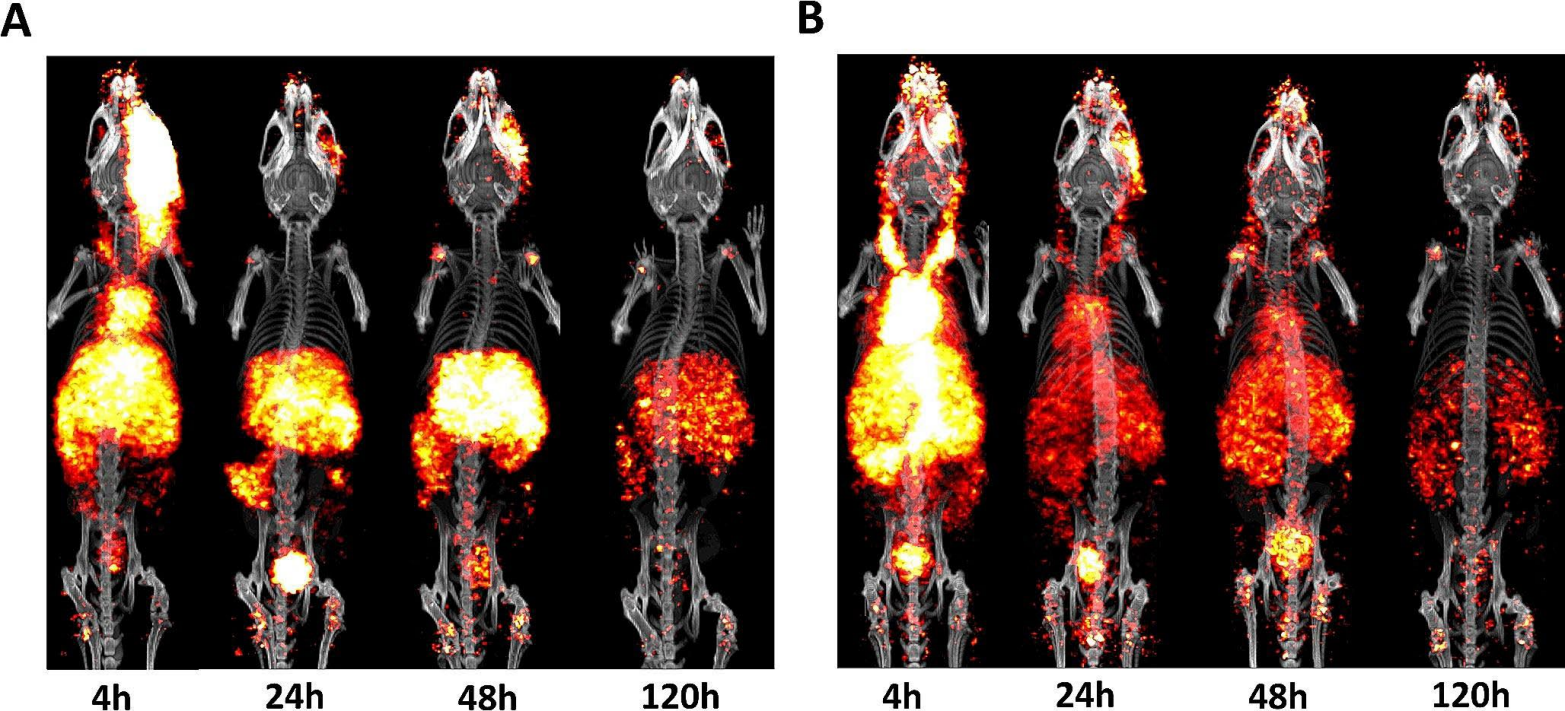
PET-CT scans of [^89^Zr]Zr-SP02SP26-ABD (**A**) and control construct [^89^Zr]Zr-R2R2-ABD (**B**) in mice with liver fibrosis. Both tracers were administered at a dose of 15 nmol/kg to mice being 3 weeks on DDC diet, and scans were acquired 4, 24, 48 and 120 h after tracer administration, while mice were still on DDC diet. Scans were corrected for injected radioactivity dose and radioactive decay, the same colorscale was applied to all corrected scans (i.e. to allow fair comparison of intensity across scans)

In the next step, the biodistribution kinetics of 15 nmol/kg of [^89^Zr]Zr-SP02SP26-ABD was evaluated in mice with overt liver fibrosis after 3 weeks of DDC intake, along with the control construct [^89^Zr]Zr-R2R2-ABD (Fig. 6). PET-CT imaging of [^89^Zr]Zr-SP02SP26-ABD at 4 h p.i. revealed high uptake in the liver region as well as blood pool activity in the cardiac region and carotids. At 24 and 48 h, uptake of [^89^Zr]Zr-SP02SP26-ABD was high and more restricted to the region of the fibrotic liver, blood pool activity was decreased, and at 120 h a decrease of the liver uptake became apparent. [^89^Zr]Zr-R2R2-ABD showed at 4 h p.i. a similar biodistribution as [^89^Zr]Zr-SP02SP26-ABD, while at later time points its distribution throughout the body was much more homogeneous and the uptake in the fibrotic liver lower (Fig. 6). The potential of [^89^Zr]Zr-SP02SP26-ABD for specific targeting of fibrotic livers was confirmed by the ex vivo biodistribution data (Supplementary Fig. 6). Liver uptake of [^89^Zr]Zr-SP02SP26-ABD was significantly higher than of [^89^Zr]Zr-R2R2-ABD at 4, 24, 48 and 120 h p.i.: 11.15 ± 0.84, 14.55 ± 0.87, 15.61 ± 0.77, and 7.56 ± 0.35%ID/g, respectively, versus 6.69 ± 0.29, 6.09 ± 0.54, 6.07 ± 0.20, and 4.53 ± 0.49%ID/g, respectively. Differences become even more striking when considering the fibrotic liver-to-blood ratios of both conjugates at 24, 48 and 120 h p.i., being 3.38 ± 0.41, 8.08 ± 0.70, and 13.03 ± 3.31, respectively, for [^89^Zr]Zr-SP02SP26-ABD and 0.48 ± 0.02, 0.76 ± 0.05, and 1.89 ± 0.50, respectively, for [^89^Zr]Zr-R2R2-ABD.

### Confirmation of PDGFRβ targeting in CCl_4_ and CDA-HFD liver fibrosis models

**Fig. 7:**
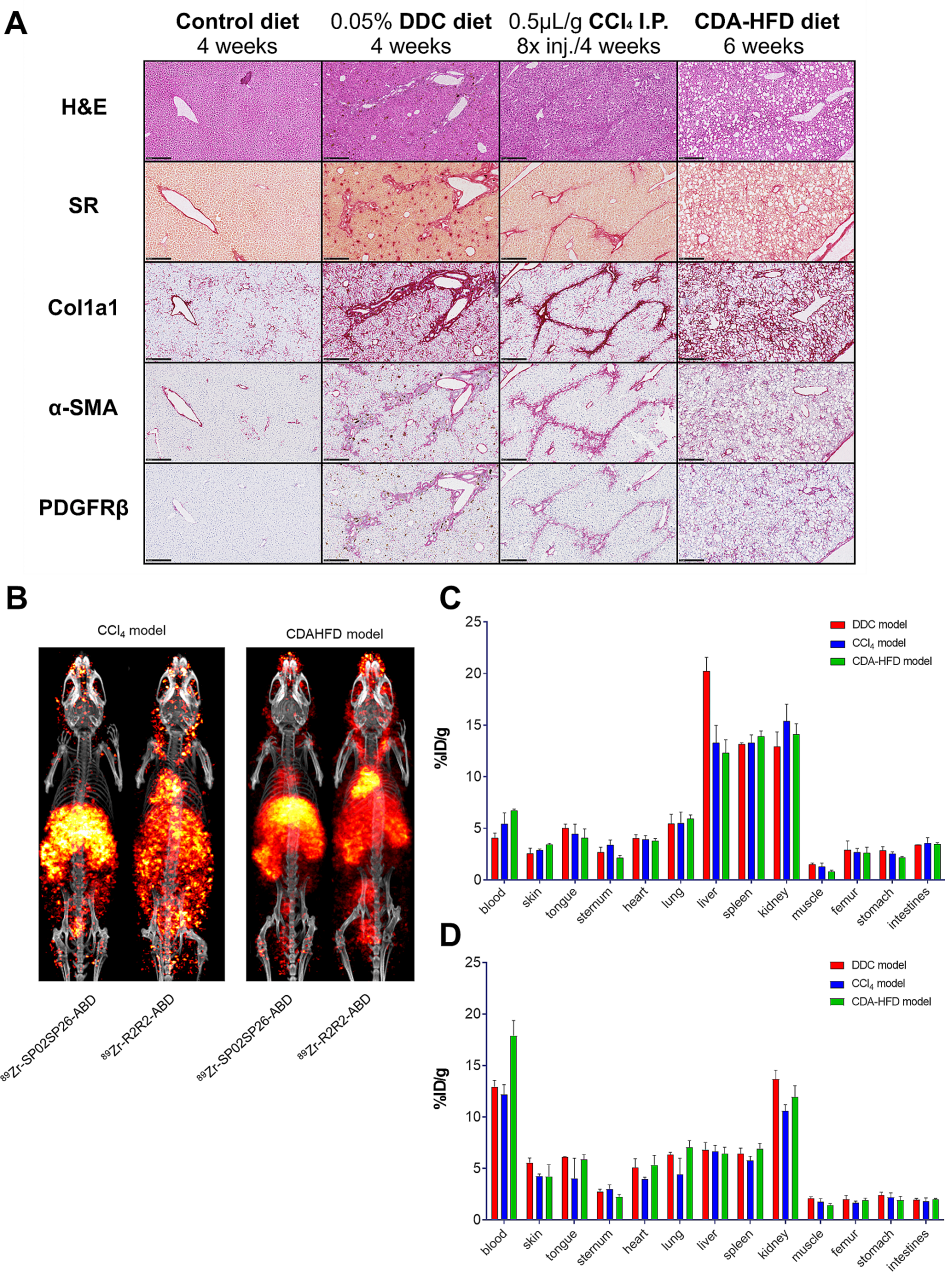
PET-CT scans and ex vivo biodistribution of [^89^Zr]Zr-SP02SP26-ABD and control construct [^89^Zr]Zr-R2R2-ABD in mice with liver fibrosis of different etiology. Liver fibrosis was induced by 4 weeks of DDC diet, 4 weeks of CCl4 treatment or 6 weeks of CDA-HFD diet. (**A**) (immuno)histochemistry of liver tissues of control mice and mice with liver fibrosis. Black bar in the bottom signifies 250 μm. Data acquired via screenshots of digital scans, using NDP.view2 software (Hamamatsu Photonics, Hamamatsu, Japan). (**B**) PET-CT scans at the end of treatment, 24 h after injection of 15 nmol/kg [^89^Zr]Zr-SP02SP26-ABD or construct [^89^Zr]Zr-R2R2-ABD. All scans were scaled to reflect the same injected ^89^Zr dose (**C**) and (**D**) As in B, ex vivo biodistribution of [^89^Zr]Zr-SP02SP26-ABD and [^89^Zr]Zr-R2R2-ABD, respectively

While the DDC model represents a cholestatic model of liver fibrosis, the carbon tetrachloride (CCl_4_) and choline-deficient, L-amino acid-defined, high-fat diet (CDA-HFD) models represent a chemotoxic and a MASLD model, respectively. To evaluate whether results observed in the DDC model are independent of the etiology of fibrosis, also the latter 2 models were characterized for expression of fibrosis biomarkers including PDGFRβ, and for targeting of fibrotic livers with [^89^Zr]Zr-SP02SP26-ABD. For this purpose, mice received 2 times weekly i.p. injections of 0.5 µL CCl_4_ per gram of body weight formulated in corn oil for 4 weeks, or a diet of CDA-HFD for 6 weeks. At the end of this period, 24 h after having received 15 nmol/kg [^89^Zr]Zr-SP02SP26-ABD or [^89^Zr]Zr-R2R2-ABD, mice were imaged by means of PET-CT and subsequently dissected for assessment of the expression of fibrosis markers and ex vivo biodistribution. As for the DDC model, for both liver fibrosis models an increased expression of fibrosis markers was observed with IHC (Fig. 7A) as well as with RT-PCR (Supplementary Table 2). PET-CT revealed increased uptake of [^89^Zr]Zr-SP02SP26-ABD in fibrotic livers in comparison with [^89^Zr]Zr-R2R2-ABD for both models (Fig. 7B). Overall, ex vivo biodistribution data revealed liver uptake levels of [^89^Zr]Zr-SP02SP26-ABD in DDC, CCl_4_ and CDA-HFD models that were higher in fibrotic livers than in control livers, and higher than for [^89^Zr]Zr-R2R2-ABD (Fig. 7C, D).

## Discussion

Based on extensive literature data, PDGFRβ might be an attractive pan-fibrosis marker with the potential to serve as an anchor for the detection and silencing of activated myofibroblasts, the drivers of fibrogenesis. To explore this possibility we designed and developed Fibrobody® SP02SP26-ABD as an anti-PDGFRβ vector primarily to be applied in Fibrobody®-Drug Conjugates for selective and effective delivery of therapeutic compounds to activated myofibroblasts.

In the present study we demonstrate that SP02SP26-ABD: (1) binds specifically to PDGFRβ, and not to PDGFRα, without activating PDGFRβ; (2) shows cross-species binding with similar nM affinity for human, mouse and rat PDGFRβ, facilitating clinical translation of preclinical findings; (3) is taken up by PDGFRβ-positive cells, which is important for intracellular drug delivery as showcased with SP02SP26-ABD-AF in vitro; (4) binds to human and murine fibrotic livers, and co-localizes to areas with increased α-SMA staining (marker for activated myofibroblasts) and collagen deposition.

Next, upon labeling of SP02SP26-ABD with ^89^Zr, [^89^Zr]Zr-SP02SP26-ABD was evaluated in 3 mouse models of liver fibrosis of different etiology: DDC, CCl_4_ and CDA-HFD model. These in vivo studies comprised the characterization of the models for fibrogenesis by analysis of fibrosis biomarkers, PET-CT imaging, assessment of ex vivo biodistribution, and autoradiography to assess intra-liver distribution of the vector. First, specific high-contrast PET-CT imaging of fibrotic livers was demonstrated with [^89^Zr]Zr-SP02SP26-ABD, while the PET-CT signal appeared related to the level of fibrogenesis and PDGFRβ expression. Second, high and selective uptake of [^89^Zr]Zr-SP02SP26-ABD in fibrotic livers was observed, up to 20%ID/g at 24 h p.i. in the DDC model, of which the largest part resulted from increased PDGFRβ expression when compared to control mice. This is remarkably high taking into account that uptake was especially confined to the fibrotic scar regions with PDGFRβ expression, as confirmed by autoradiography. While the first observation is promising from a diagnostic perspective, the second observation is important from a therapeutic perspective, and combined both hold great promise for exploring Fibrobody® SP02SP26-ABD in a theranostic approach for liver fibrosis and potentially other types of fibrosis as well.

To rank the suitability of SP02SP26-ABD as a vector for drug delivery, we estimated the number of SP02SP26-ABD molecules that can be delivered to each PDGFRβ-positive cell in the DDC model (at the 4 week time point). Assuming a mouse body weight of 25 g; saturation of PDGFRβ at ∼ 20 nmol/kg SP02SP26-ABD corresponding to ∼ 0.5 nmol/mouse; increased liver uptake of SP02SP26-ABD due to increased PDGFRβ expression: 13.37%ID/g (Fig. 4); percentage of PDGFRβ-positive cells in the liver: ∼25% (Fig. 3); residualization of ^89^Zr: 100%; number of cells per gram of liver: 10^9^; Number of Avogadro: 6.02 × 10^23^. This estimation reveals the delivery of ∼ 160,000 SP02SP26-ABD molecules per PDGFRβ-positive cell, which is considered a substantial number for antibody-drug conjugate approaches.

During the course of our studies, two studies were published on the use of small molecular ^18^F- and ^68^Ga-labeled anti-PDGFRβ Affibody constructs (∼ 6.5 and 15 kDa) for PET imaging of liver fibrosis [36, 37]. Although having high affinity for PDGFRβ, and showing a similar spatial binding pattern on human and murine fibrotic liver tissues as [^89^Zr]Zr-SP02SP26-ABD, the renal clearance of these PET tracers was extremely rapid, resulting in high kidney uptake levels and low uptake levels in fibrotic livers. Despite this, using the 15 kDa radiolabeled dimerized affibody [^68^Ga]Ga-DOTA-Z_PDGFRß_ in a 4 weeks CCl4 treatment model, a liver-to-blood ratio of 3 was already obtained 2 h post injection, showing the potential of this construct for diagnostic purposes [37]. Rapid renal clearance and excessive kidney uptake of these targeting vectors can be explained by the molecular weight being below the glomerular filtration threshold. The fact that these phenomena were not observed with [^89^Zr]Zr-SP02SP26-ABD is due to the presence of an ABD unit.

Next to increased liver uptake of [^89^Zr]Zr-SP02SP26-ABD during liver fibrogenesis, we also observed relatively high spleen and kidney uptake in healthy mice, an observation which can be explained by physiological PDGFRβ expression in these organs. Whether targeting of these normal tissues will hamper the clinical application of Fibrobody® Drug Conjugates is matter of speculation at this moment, and might strongly depend on the preference and mode of action of the delivered drug. Ideally, the drug silences PDGFRβ-expressing activated myofibroblasts, while keeping non-fibrotic PDGFRβ-expressing cells unaffected. As an initial step to test tolerability, we have treated healthy mice with a saturating dose of 135 nmol/kg SP02SP26-ABD-AF, and such treatment did not result in unacceptable weight loss or histological alterations (data not shown). It is of note that the dose of auristatinF tested in these studies was well beyond a level known to cause anti-tumor effects [38].

Based on the studies described herein, clinical PET-CT imaging studies with [^89^Zr]Zr-SP02SP26-ABD will be started shortly. These studies will not be restricted to patients with advanced stage liver fibrosis, but also include types of fibrosis in which PDGFRβ-expressing myofibroblasts are assumed to play a key role, such as lung fibrosis and kidney fibrosis. These studies will learn whether PDGFRβ is a suitable anchor and SP02SP26-ABD a suitable pan-fibrosis vector for selective targeting of active fibrosis. In the meantime, preclinical efforts will be expanded to design SP02SP26-ABD-based drug conjugates optimally equipped to silence and revert active fibrosis in an effective, selective and safe way.

## Electronic supplementary material

Below is the link to the electronic supplementary material.

Supplementary Material 1

## Data Availability

Data to support the findings in this study are included in the manuscript or its supplementary information. Additional data can be made available upon reasonable request from the corresponding author (vandongen@linxispharmaceuticals.nl).
