# Supplementary material for: Preclinical targeting of liver fibrosis with a 89Zr-labeled Fibrobody® directed against platelet derived growth factor receptor-β
Source: Eur J Nucl Med Mol Imaging. 2024 Jun 18;51(12):3545–58. doi: 10.1007/s00259-024-06785-9 (PMC11445362; doi:10.1007/s00259-024-06785-9)
Supplement: Supplementary file 1 — Supplementary Material 1 [file 259_2024_6785_MOESM1_ESM.docx]

**Supplementary information**

**Fibrobody® SP02SP26-ABD and control construct** **R2R2-ABD**

VHHs directed against human PDGFRβ were obtained after immunization of two llamas at Eurogentec (Seraing, Belgium) with four injections of human PDGFRβ protein ectodomain (25 μg per injection; Sino-Biological, Eschborn, Germany) with intervals of 2 weeks. Four weeks after the last interval, a final boost injection with SCC-hPDGFRβ (10^8^ cells) was administered. For rat PDGFRβ, a similar approach was followed, using rat PDGFRβ ectodomain and SCC-rPDGFRβ instead [1]. Phage selection, isolation and characterization of SP02 and SP26, and construction of the final species cross-reactive and internalizing SP02SP26-ABD construct, will be described in detail elsewhere. We used the bivalent nanobody construct R2R2-ABD, directed against azo dye Reactive Red as a non-binding control [2,3]. Both constructs contain a near C-terminal cysteine for conjugation purposes [4].

The VHH constructs were produced and purified by ImmunoPrecise Antibodies (Utrecht, The Netherlands) following established protocols, and obtained as a mixture of monomeric and dimeric (via disulfide bond) proteins. Briefly, VHH constructs were produced in HEK293E-253 mammalian cells through transfection with plasmid DNA. Six days post-transfection, the culture medium containing the VHH constructs was harvested using a low-speed centrifugation step. Purification of the VHH constructs was performed using the EPEA tag and gel filtration. The fractions containing the VHH constructs were subjected to LabChip capillary electrophoresis to confirm their purity and a LAL assay to determine endotoxin levels. To ensure sterility, the VHH constructs were subsequently filtered through a 0.22 μm syringe filter.

**Effect of Fibrobody®** **SP02SP26-ABD on PDGFRβ activation**

To investigate whether the SP02SP26-ABD construct possesses agonistic activity, phosphorylation of PDGFRβ was checked by means of Western blotting. SCC-hPDGFRβ cells were serum starved for 4 hrs followed by an incubation of 15 min with SP02SP26-ABD-ethyl. As a positive control for PDGFRβ activation, the natural PDGFRβ ligand platelet derived growth factor BB (PDGF-BB) was used. After incubation, cells were lysed in SDS-PAGE loading buffer. Phosphorylated PDGFRβ was detected using a rabbit anti-pPDGFRβ antibody Tyr751 (3161S, Cell Signaling Technology, Leiden, The Netherlands) followed by an IRDye800CW-conjugated anti-rabbit antibody (926-32211, Li-COR Biosciences, Bad Homburg, Germany). Loading was checked using a mouse anti-Tubulin antibody (DM1A, 3873S, Cell Signaling Technology, Leiden, The Netherlands), followed by an Alexa Fluor 647-conjugated anti-mouse antibody (A-21235, ThermoFisher Scientific, Bleiswijk, The Netherlands).

**IRDye800CW conjugation to VHH constructs**

**Scheme S1:** SP02SP26-ABD-IRDye800CW

**
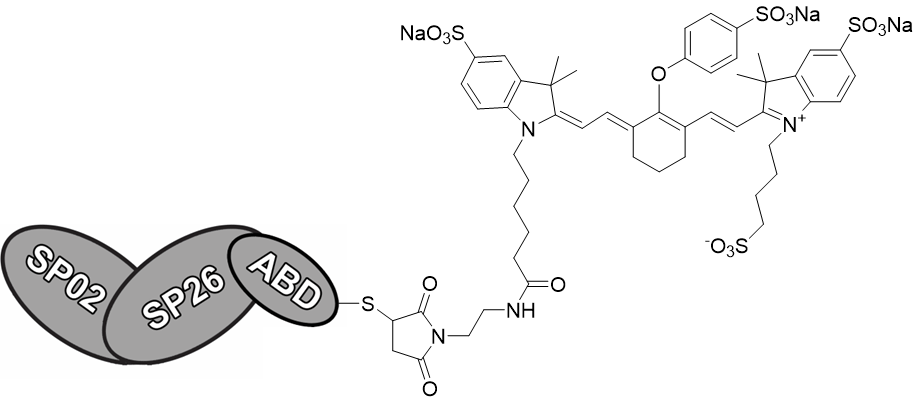
**

The near C-terminal cysteine in the SP02SP26-ABD and R2R2-ABD constructs was used for site-specific maleimide-IRDye800CW (LI-COR Biosciences, Lincoln, NE, USA) conjugation as described previously [4]. Briefly, VHH constructs were incubated with 2 molar equivalents of tris(2-carboxyethyl)phosphine hydrochloride (TCEP.HCl) in borate buffer (25 mM sodium borate pH 8, 25 mM NaCl, 1 mM DTPA) at 37 °C for 2 hrs. The maleimide-IRDye800CW was added at 5 molar equivalents and incubated on ice for 1 h. The conjugates were purified from unbound fluorophore using two consecutive 2 mL Zeba spin desalting columns (Thermo Fisher Scientific, Bleiswijk, The Netherlands), which were equilibrated with PBS. The amount of unbound dye in the samples was determined by SDS-PAGE, and fluorescence was detected with the Typhoon™ imager (Cytiva, Medemblik, The Netherlands). The degree of labeling was determined following the manufacturer’s protocol by measuring the absorbance at 280 and 800 nm using a Nanodrop spectrophotometer (Nanodrop Technologies, Wilmington, DE, USA). The degree of labeling for both constructs was near 1, qualifying them for further analysis.

**AuristatinF** **conjugation to VHH constructs**

**Scheme S2:** SP02SP26-ABD-AF

**
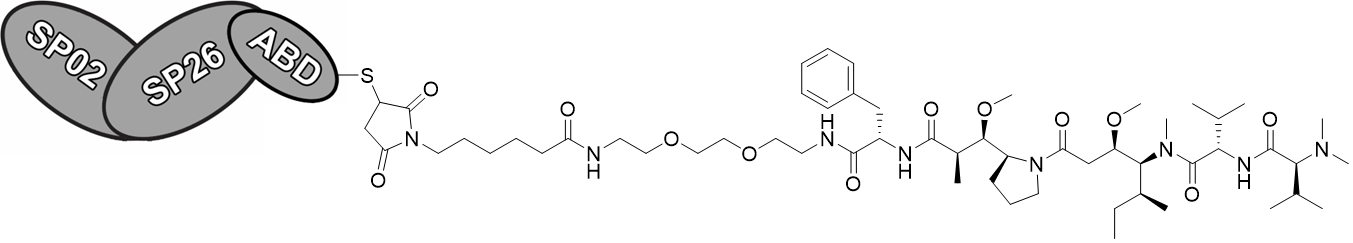
**

The near C-terminal cysteine in the VHH constructs was used for site-specific N-(2-(2-(2-AF-amidoethoxy)ethoxy)ethyl)-6-(2,5-dioxo-2,5-dihydro-1H-pyrrol-1-yl)hexanamide (maleimide-auristatinF) conjugation [5]. Briefly, VHH constructs were incubated with 2 molar equivalents of TCEP.HCl in borate buffer (25 mM sodium borate pH 8, 25 mM NaCl, 1 mM DTPA) at 37°C for 2 hrs. The maleimide-auristatinF (10 mM solution in DMSO) was added at 5 molar equivalents and incubated on ice for 1 h. The reaction products were purified by Amicon® Ultra Centrifugal Filters (10 kDa molecular weight cut-off spin filters, 4x wash with PBS). After purification, the products were reconstituted in PBS to the desired volume and a sample was obtained for HPL-SEC to determine protein integrity.

**Ethyl conjugation of VHH constructs**

**
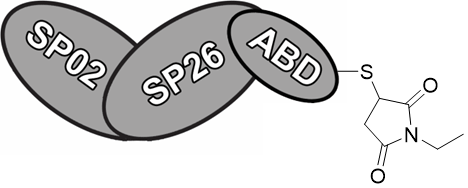
Scheme S3:** SP02SP26-ABD-ethyl

The near C-terminal cysteine in the VHH constructs was used for site-specific conjugation of maleimide-ethyl (Merck, Darmstadt, Germany). Briefly, VHH constructs were incubated with 2 molar equivalents of TCEP.HCl in borate buffer (25 mM sodium borate pH 8, 25 mM NaCl, 1 mM DTPA) at 37°C for 2 hrs. The maleimide-ethyl (10 mM solution in DMSO) was added at 5 molar equivalents and incubated on ice for 1 h. The reaction products were purified by Amicon® Ultra Centrifugal Filters (10 kDa molecular weight cut-off spin filters, 4x wash with PBS). After purification, the products were reconstituted in PBS to the desired volume and a sample was obtained for HPL-SEC to determine protein integrity.

**DFO* conjugation to VHH constructs**

**Scheme S4:** SP02SP26-ABD-DFO*

**
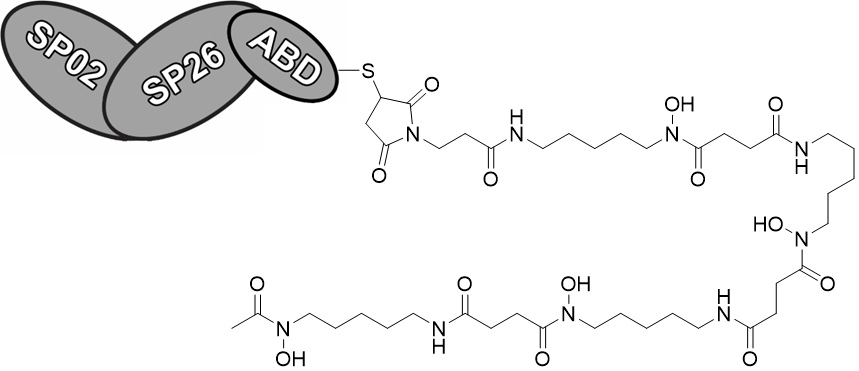
**

The near C-terminal cysteine in the VHH constructs was used for site-specific N^1^-(1-(2,5-dioxo-2,5-dihydro-1H-pyrrol-1-yl)-10,21-dihydroxy-3,11,14,22,25-pentaoxo-4,10,15,21,26-pentaazahentriacontan-31-yl)-N^1^-hydroxy-N^4^-(5-(N-hydroxyacetamido)pentyl)succinimide (maleimide-DFO*, ABX, Radeberg, Germany) conjugation. Briefly, VHH constructs were incubated with 2 molar equivalents of TCEP.HCl in borate buffer (25 mM sodium borate pH 8, 25 mM NaCl, 1 mM DTPA) at 37 °C for 2 hrs. The maleimide-DFO* (5 mM solution in DMSO) was added at 5 molar equivalents and incubated on ice for 1 h. The reaction products were subsequently purified by PD-10 Desalting Columns contain Sephadex G-25 (GE17-0850-01, Sigma Aldrich; 0.9% NaCl eluent, according to manufacturer’s instructions) followed by spin filtration (10 kDa molecular weight cut-off spin filters, 4x wash with 0.9% NaCl). After purification, the products were reconstituted in 0.9% NaCl to the desired volume and a sample was obtained for HPL-SEC to determine protein integrity and for ELISA to test antigen binding.

**Binding to PDGFRβ ectodomain and SCC-PDGFRβ cells**

Recombinant human, mouse or rat PDGFRβ ectodomain (Sino-Biological, Eschborn, Germany) at 1 μg/ml was coated overnight at 4 °C in ELISA plates (Nunc MaxiSorp™, Thermo Fisher Scientific, Bleiswijk, The Netherlands). The next day, plates were washed with PBS and blocked with blocking buffer (PBS, supplemented with 1% (w/v) BSA) for 1 h at RT. Next, five-fold serial dilutions of DFO*- or IRDye800CW-labeled VHH constructs were added in PBS starting from 200 nM. All incubations were carried out for 1 h at RT, and after every incubation plates were washed four times with PBS containing 0.05% Tween-20. DFO*-conjugates were detected using a rabbit anti-VHH polyclonal antibody (QVQ, Utrecht, The Netherlands), followed by an IRDye800CW-conjugated anti-rabbit antibody (926-32211, Li-COR Biosciences, Bad Homburg, Germany). After the final wash, 100 μL PBS was added to each well, and plates were read out on a Odyssey near-infrared fluorescence scanner (LI-COR Biosciences, Lincoln, NE, USA). Results were analyzed with Graphpad Prism software (using the one-site specific binding, non-linear fit function of fluorescence versus (log) conjugate concentration). For binding to cells, a similar procedure was followed. In short, 5000 cells were seeded out in cell culture treated flat bottom 96-well plates (Nunclon Delta Surface, ThermoFisher Scientific, Bleiswijk, The Netherlands). After two days, four-fold serial dilutions, prepared in DMEM without phenol red supplemented with 25mM Hepes and 1% BSA, starting from 100 nM of IRDye800CW-labeled VHH constructs were added to the cells. After 2 hrs of incubation on ice, cells were washed three times. After the final wash, 100 μl PBS was added to the wells. Plate read-out and data analysis was done as described above.

**^89^Zr-labeling and quality tests**

^89^Zr radiolabeling was performed as described before [6]. In short, an acidic solution of ^89^Zr in 1M oxalic acid (Revvity, Waltham, MA, USA) was neutralized by addition of 2M Na_2_CO_3_ followed by addition of an excess of HEPES buffer (pH 7.5). The DFO*-conjugated VHH constructs were then added to the mixtures. After 60 min the reaction mixture was purified on a prewashed PD-10 column, yielding the ^89^Zr radiolabeled VHH constructs.

Protein integrity (i.e. monomeric purity) and radiochemical integrity (i.e. integrity of the radiolabeled VHH constructs as individual, radiolabeled monomers) was assessed by means of HPL-SEC using a Shimadzu HPLC system equipped with a Sepax Zenix-C SEC-300 column (300 Å, 7.8 × 300 mm) (Sepax Technologies Inc., Newark, DE, USA) as the solid phase, and an aqueous phosphate buffer as the mobile phase (conform column manufacturer recommendation). Detection was performed by absorbance at 280nm and radio-detector. Monomeric purity of all radiolabeled conjugates was >95%.

Radiochemical purity was assessed by instant thin-layer chromatography (iTLC) analysis, using silica-impregnated glass fibersheets (PI Medical Diagnostic Equipment BV, Raamsdonkveer, The Netherlands) as the solid phase and 20 mmol/L citrate buffer (pH 5.0)/MeCN (7:3) as the mobile phase. Radiochemical purity was defined as percentage of ^89^Zr area bound to protein vs total ^89^Zr area. Detection of protein bound and unbound fractions was performed on a Hidex Automatic Gamma Counter. Radiochemical purity of all radiolabeled conjugates was >95%.

*In vitro* binding characteristics of [^89^Zr]Zr-SP02SP26-ABD were determined in an immunoreactivity assay, essentially as described by Lindmo *et al* [7], using a serial dilution of 0.2% glutaraldehyde-fixed SCC-hPDGFRβ cells (concentration range from 1.25x10^6^ cells/mL to 7.8x10^4^ cells/mL) and a fixed amount of [^89^Zr]Zr-SP02SP26-ABD (0.468 pmol/mL). After incubation at 4 °C overnight, the cell suspension was centrifuged and the specific binding was calculated as the ratio of cell-bound radioactivity to the total amount of radioactivity in the assay. This was corrected for non-specific binding, as determined with a 427-fold excess of non-radiolabeled SP02SP26-ABD. Binding assays were performed in triplicate. The immunoreactive fraction of all radiolabeled PDGFRβ-targeting conjugates was >90%.

**(Immuno)histochemistry**

Animal tissues used in (immuno-)histochemistry were flash-frozen in liquid nitrogen cooled isopentane, after which sectioning was performed using a cryostat (Leica CM3050 S). Briefly, tissues were mounted using tissue freezing medium (14020108926, Leica Biosystems). 8 and 14 μm cryosections were cut at temperatures between -15 °C and -25 °C, mounted onto glass object slides, air dried for at least several min at RT, and then stored at -80 °C until further processing. 8 μm sections were used for immunohistochemistry and hematoxylin-eosin staining, 14 μm sections for (picro)sirius red staining.

For H&E staining, sections were first fixated in 2% phosphate-buffered formaldehyde (P6148, Merck, Amsterdam, The Netherlands) for 10 min, after which they were washed 3 times with demineralized water. Then, slides were stained with Mayers’ haematoxylin (MHS32, Merck, Amsterdam, The Netherlands) for 10 min, followed by bluewing in tap water and a 2 min stain with acidified aqueous eosin Y solution (HT110216, Merck, Amsterdam, The Netherlands) acidified with 0.5% v/v acetic acid. Slides were then washed and dehydrated, after which they were mounted using permanent mounting medium (Shandon-Mount, 12825443, Epredia, Breda, The Netherlands).

For (picro)sirius red staining, sections were first fixated in 2% phosphate-buffered formaldehyde (P6148, Merck, Amsterdam, The Netherlands) for 10 min at RT, after which they were washed 3 times with PBS. Then, slides were stained with (picro)sirius red solution (0,1% w/v Direct red 80 (365538, Merck, Amsterdam, The Netherlands) in 1.3% v/v picric acid (P6744, Merck, Amsterdam, The Netherlands)) for 45 min, followed by 2 washes in 0.5% acetic acid (v/v in demineralized water, A6283, Sigma-Aldrich) for 2 min each. Slides were then washed and dehydrated, after which they were mounted using permanent mounting medium (Shandon-Mount, 12825443, Epredia, Breda, The Netherlands).

For immunohistochemistry, sections were first fixated in 2% phosphate-buffered formaldehyde (P6148, Merck, Amsterdam, The Netherlands) for 10 min at RT, after which they were washed 3 times with PBS. Then, slides were blocked using the HRP/AP blocking reagent (SP-6000, Vector Laboratories Inc, Newark, CA, USA) for 5 min and washed. Sections were subsequently stained overnight with relevant primary antibodies, washed, incubated with AP-conjugated secondary antibody (DPVR55AP, Immunologic, Arnhem, The Netherlands) for 2 h at 37 ^o^C, and washed. Primary antibodies used: anti-PDGFRβ (28E1, monoclonal rabbit IgG, 1/100 diluted, Cell Signaling, Leiden, The Netherlands); anti-α-SMA (used in human tissues, D4K9N, monoclonal rabbit IgG, 1/500 diluted, Cell Signaling, Leiden, The Netherlands); anti-α-SMA (used in mouse tissues, EPR5368, monoclonal rabbit IgG, 1/35.000 diluted, Abcam, Cambridge, UK); anti-Col1A1 (E8F4L, monoclonal rabbit IgG, 1/200 diluted, Cell Signaling). All listed antibodies were diluted in Brightdiluent, normal antibody diluent (BD09, Immunologic, Arnhem, The Netherlands).

Finally, to develop the slides, sections were covered with freshly prepared AP-substrate solution (76713, Cell Signaling, Leiden, The Netherlands), incubated for 20 min at RT in a humidified slide chamber. Staining development was stopped by means of a wash in an abundance of PBS. Afterwards, slides were counterstained with Mayers’ haematoxylin (MHS32, Merck, Amsterdam, The Netherlands). Slides were then washed and dehydrated, after which they were mounted using permanent mounting medium (Shandon-Mount, 12825443, Epredia, Breda, The Netherlands).

Immunohistochemistry to assess VHH construct binding was performed with a modified protocol of immunohistochemistry staining as described above. To avoid albumin binding, for this purpose SP02SP26-ethyl and R2R2-ethyl (serving as a non-binding VHH) were used instead of their ABD-containing counterparts. After thawing, sections were immediately incubated with construct SP02SP26-ethyl at 25 nM for 30 min at 37 °C, after which they were washed 3 times with 0.05% Tween 20 in PBS. Next, sections were fixated in 2% phosphate-buffered formaldehyde for 10 min at RT, after which the protocol was continued the same as the previously described protocol (from HRP/AP blocking and onwards), using an anti-VHH mAb (96A3F5 1/2000 diluted, GenScript, Rijswijk, The Netherlands) as a primary antibody for detection of any bound SP02SP26-ethyl or R2R2-ethyl.

All above slides were digitized by means of a slide scanner at the Pathology Laboratory of University Medical Centre of Utrecht (UMCU).

**RT-PCR**

Total RNA was extracted from frozen liver sections using Trizol reagent (ThermoFisher Scientific, Bleiswijk, The Netherlands). The first-strand cDNA was reverse transcribed from the total RNA using the High-Capacity cDNA Reverse Transcription Kit (ThermoFisher Scientific, Bleiswijk, The Netherlands). Then, real-time PCR was performed using Power SYBR™ Green PCR Master Mix (ThermoFisher Scientific, Bleiswijk, The Netherlands). Primer sequences used are shown in supplementary Table 1. Data were analyzed using the 2^−ΔΔCT^ method with GAPDH serving as an internal control. The healthy control animals served as reference group in the study.

**Serum markers for liver damage**

Individual blood samples were centrifuged for 10 min at 5000g. After centrifugation, serum was transferred to a new tube and placed at -80°C until analysis. Individual sera were analyzed at the Central Diagnostical Laboratory of the Amsterdam University Medical Center using their clinical robotics system for levels of Alkaline Phosphatase (AP), Alanine aminotransferase (ALT) and Aspartate aminotransferase (AST). The robotics system requires a minimum sample volume of 150-200 μL. To accommodate, all samples were diluted 2-fold in saline before analysis. To obtain final AP and ASAT results, the measurements were first corrected for sample hemolysis (using the calibration data from the Central Diagnostical Laboratory).

**Supplementary Table 1**: RT-PCR primer sequences

| **Transcript** | **Forward primer (5’ - > 3’)** | **Reverse primer (5’ - > 3’)** |
| --- | --- | --- |
| *Gapdh* | CCTTCATTGACCTCAACTACATG | TCTCGCTCCTGGAAGATGGTG |
| *Pdgfrβ* | CCCCTTACCCAGAGCTGCC | TCTGCATGATCTCATAGATCTCG |
| *Col1a1* | GAGAGCATGACCGATGGATTCC | TAGGTGATGTTCTGGGAGGCC |
| *Col1a2* | CTCCTGAAGGCTCTAGAAAGAAC | CATCCTTGGTTAGGGTCAATCC |
| *Col3a1* | ATTTAGACATGATGAGCTTTGTGC | GGTTCTGGCTTCCAGACATCTC |
| *Desmin* | GAGATGGCCCGCCATCTGCG | GGCTGGTTTCTCGGAAGTTGAG |
| *α-Sma/Acta2* | AGTGTGATATTGACATCAGGAAGG | CTGATCCACATCTGCTGGAAGG |
| *Pdgfrα* | GCAAGAGGAACAGACACAGCTC | GGTCTCGTCCTCTCTCTTGATG |
| *Timp1* | TAAGGAACGGAAATTTGCACATC | TGTCACTCTCCAGTTTGCAAGG |
| *Lox* | GCAGACATAGACTGCCAGTGG | CCAGGTAGCTGGGGTTTACAC |
| *Tgfβ1* | TGGAAGTGGATCCACGAGCCC | CCTGCGGCACGCAGCACGG |

**Supplementary Table** **2** Expression of fibrosis related genes as assessed by RT-PCR in liver tissues of healthy mice or of mice treated with CCl_4_ for 4 weeks or fed with CDA-HFD diet for 6 weeks. -: not upregulated, +: 1-5 fold upregulated, ++: 5-20 fold upregulated, +++>20 fold upregulated in mRNA isolated from liver homogenates, as compared to healthy controls.

| **Transcript** | **4 weeks**  **CCl4** | **6 weeks**  **CDA-HFD** |
| --- | --- | --- |
| Pdgfrβ | + | ++ |
| Col1a1 | ++ | +++ |
| Col1a2 | + | +++ |
| Col3a1 | + | +++ |
| Desmin | + | + |
| α-Sma | + | + |
| Pdgfrα | + | ++ |
| Timp1 | + | +++ |
| Lox | + | +++ |
| Tgfβ1 | + | ++ |


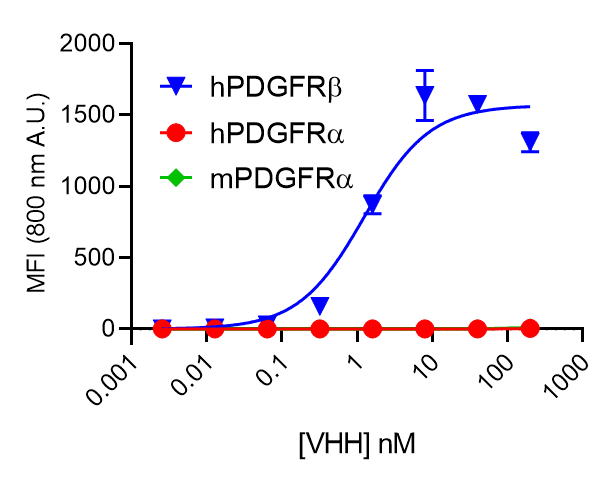

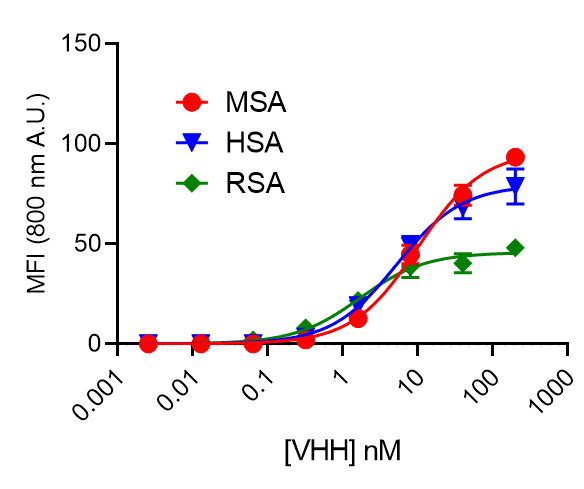
**Supplementary Fig. 1** Binding of SP02SP26-ABD-IRDye800CW to mPDGFRα and hPDGFRα ectodomain. As a control for binding, hPDGFRβ ectodomain was taken along. MFI = Median Fluorescence Intensity

**Supplementary Fig. 2** Binding of SP02SP26-ABD-IRDye800CW to human (HSA), mouse (MSA), and rat serum albumin (RSA). MFI = Median Fluorescence Intensity.


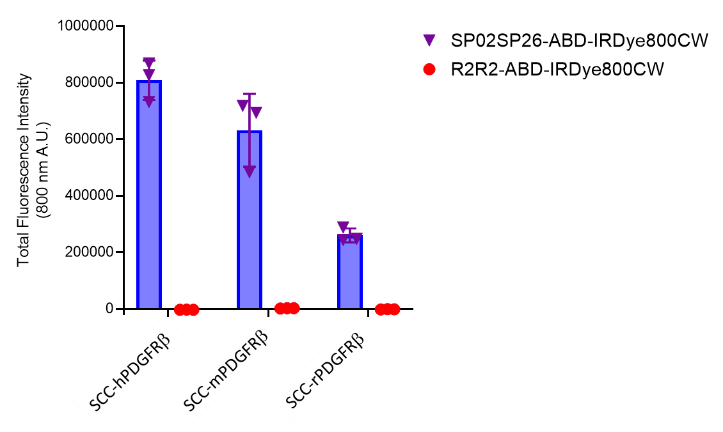


**Supplementary Fig. 3** Uptake of SP02SP26-ABD-IRDye800CW and R2R2-ABD-IRDye800CW after 60 min incubation by SCC-hPDGFRβ, SCC-mPDGFRβ and SCC-rPDGFRβ cells.

**
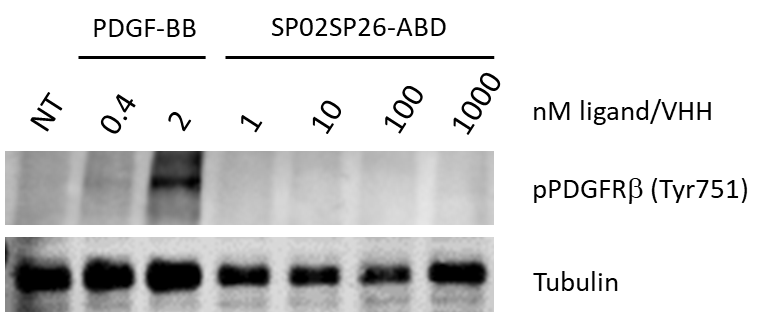
**

**Supplementary Fig. 4** Effect of SP02SP26-ABD on PDGFRβ phosphorylation using SCC-hPDGFRβ cells. Western blot of phosphorylated PDGFRβ after PDGF-BB treatment and after SP02SP26-ABD treatment.


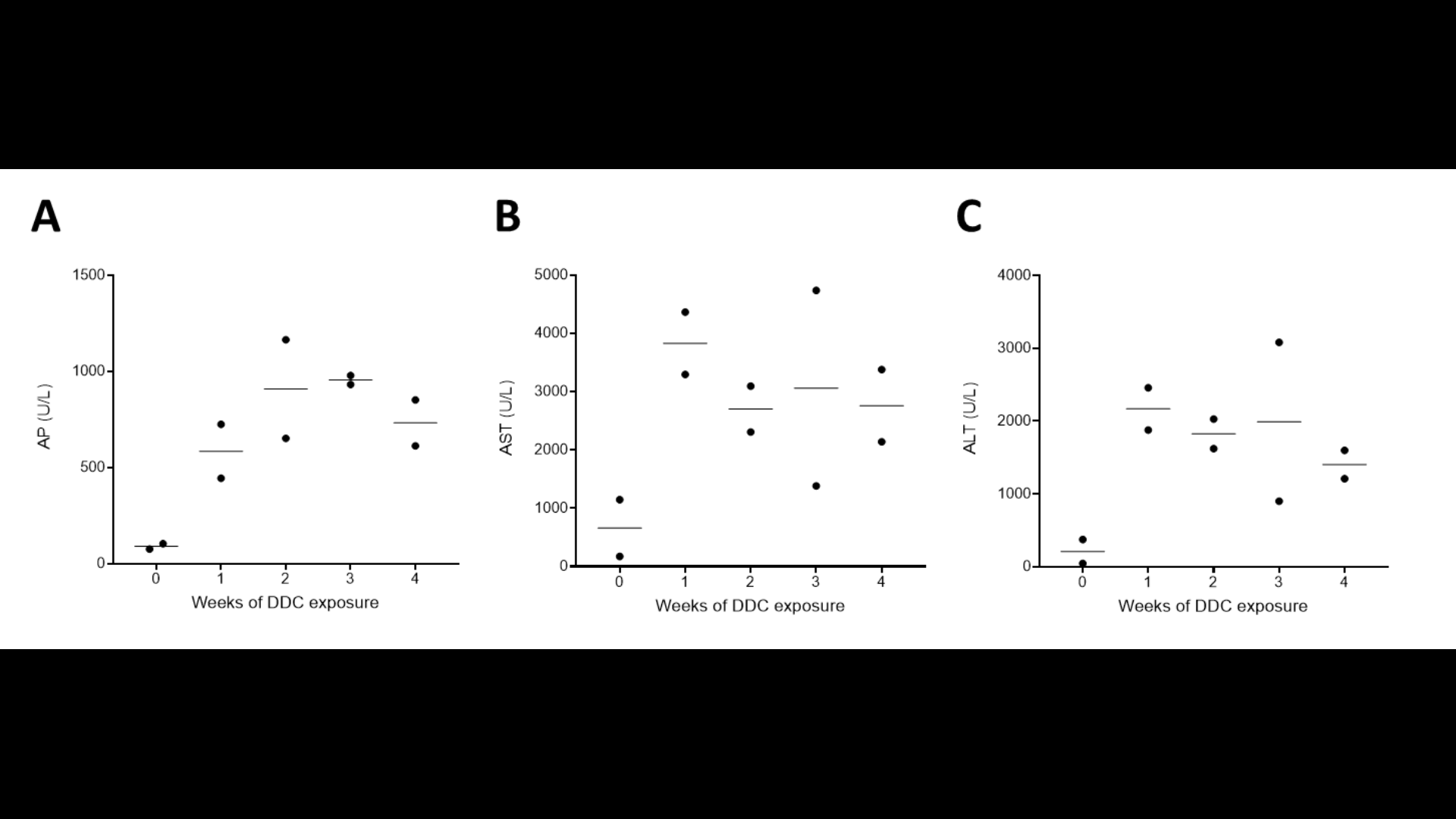


**Supplementary Fig. 5** Serum markers for liver damage of mice at different time intervals on DDC diet. A: AP (Alkaline phosphatase). B: AST (Aspartate aminotransferase). C: ALT (Alanine aminotransferase).


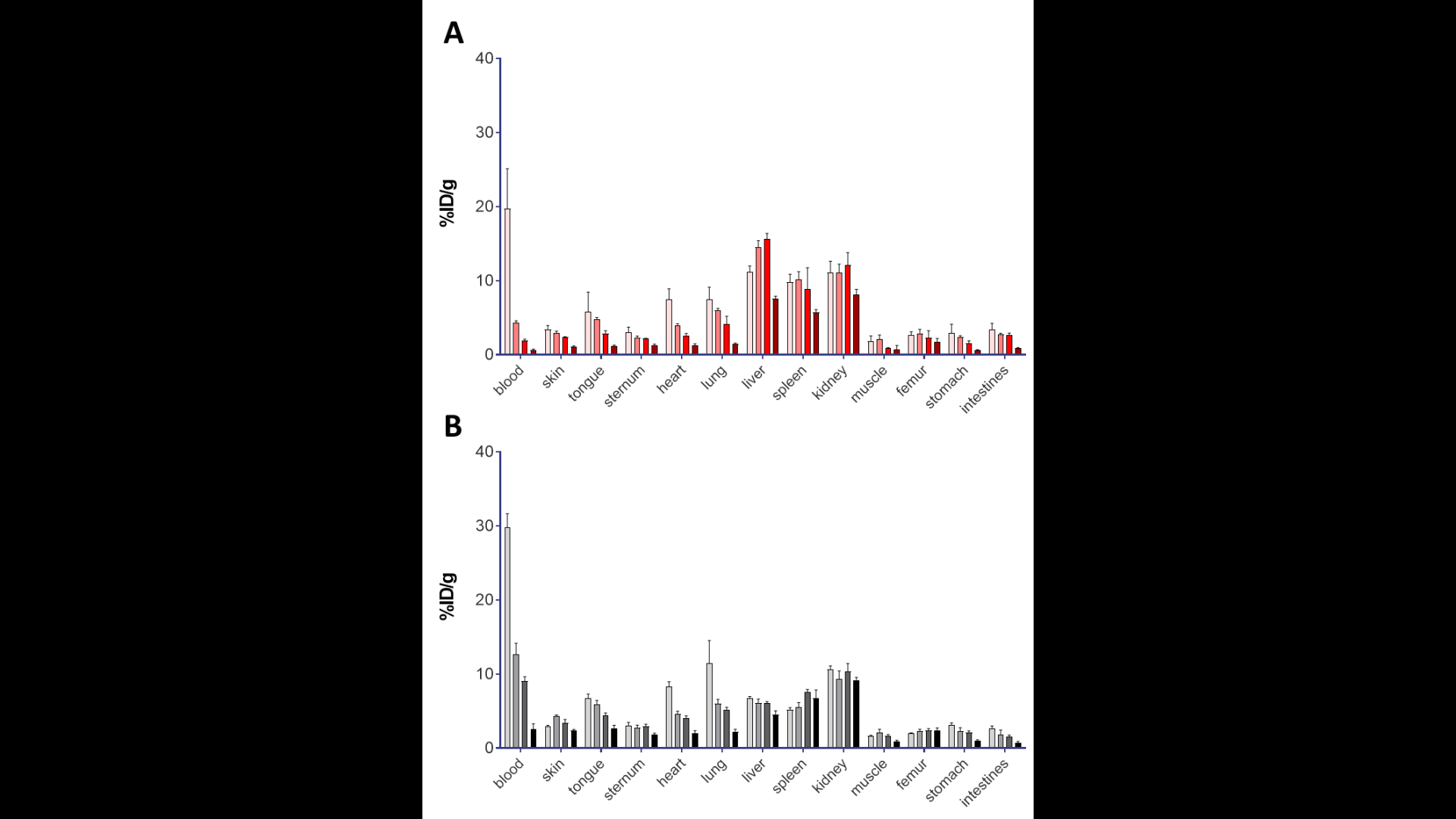


**Supplementary Fig 6** *Ex vivo* biodistribution of [^89^Zr]Zr-SP02SP26-ABD directed against PDGFRβ **(A)** or control [^89^Zr]Zr-R2R2-ABD **(B)** in mice with liver fibrosis. Both tracers were administered at a dose of 15 nmol/kg after being 3 weeks on DDC diet (n=3 per group), and *ex vivo* biodistribution was assessed 4, 24, 48 and 120 hrs after tracer administration, while mice were still on DDC diet. Uptake levels are expressed as the average percentage of the injected (activity-) dose per gram of tissue (%ID/g) plus SD.

**References**

1. Pronk SD, Schooten E, Heinen J, Helfrich E, Oliveira S, Van Bergen en Henegouwen PMP. Single domain antibodies as carriers for intracellular drug delivery: a proof of principle study. Biomolecules 2021;11:927. Doi: 10.3390/biom11070927.
2. Frenken LG, van der Linden RH, Hermans PW, Bos JW, Ruuls RC, de Geus B, et al. Isolation of antigen specific llama VHH antibody fragments and their high level secretion by Saccharomyces cerevisiae. J. Biotechnol. 2000;78:11-21. doi:10.1016/s0168- 1656(99)00228-x
3. Oliveira S, Van Dongen GAMS, Stigter-van Walsum M, Roovers RC, Stam JC, Mali W, et al. Rapid visualization of human tumor xenografts through optical imaging with a near-infrared fluorescent anti-epidermal growth factor receptor nanobody. Mol Imaging 2012;11:33-46. doi:10.2310/7290.2011.00025.
4. Kijanka M, Warnders F-J, El Khattabi M, Lub-de Hooge M, Van Dam GM, Ntziachristos V, et al. Rapid optical imaging of human breast tumour xenografts using anti-HER2 VHHs site-directly conjugated to IRDye 800CW for image-guided surgery. Eur. J. Nucl. Med. Mol. Imaging 2013;40:1718-29. Doi: 10.1007/s00259-013-2471-2.
5. Sijbrandi NJ, Merkul E, Muns JA, Waalboer DC, Adamzek K, Bolijn M, et al. A novel platinum(II)-based bifunctional ADC linker benchmarked using ^89^Zr-desferal and auristatin F conjugated trastuzumab. Cancer Res. 2017;77:257-67. doi 10.1158/0008-5472.CAN-16-1900.
6. Van Dongen GAMS, Beaino W, Windhorst AD, Zwezerijnen GJC, Oprea-Lager DE, Hendrikse NH, et al. The role of ^89^Zr-immuno-PET in navigating and derisking the development of biopharmaceuticals. J. Nucl. Med. 2021;62:438-45. doi:10.2967/jnumed.119.239558.
7. Lindmo T, Boven E, Cuttitta F, Fedorko J, Bunn Jr. PA. Determination of the immunoreactive fraction of radiolabeled monoclonal antibodies by linear extrapolation to binding at infinite antigen excess. *J. Immunol. Methods*. 1984;72:77-89. doi:10.1016/0022-1759(84)90435-6.
